# Migrasome Marker Epidermal Growth Factor Domain-Specific *O*-GlcNAc Transferase: Pan-Cancer Angiogenesis Biomarker and the Potential Role of circ_0058189/miR-130a-3p/EOGT Axis in Hepatocellular Carcinoma Progression and Sorafenib Resistance

**DOI:** 10.3390/biomedicines13040773

**Published:** 2025-03-22

**Authors:** Zhe Yu, Jing Luo, Wen An, Herui Wei, Mengqi Li, Lingling He, Fan Xiao, Hongshan Wei

**Affiliations:** 1 Department of Gastroenterology, Peking University Ditan Teaching Hospital, Beijing 100015, China; yuzhe@pku.edu.cn (Z.Y.); 2211110783@pku.edu.cn (J.L.); 2 Department of Cancer Center, Beijing Ditan Hospital, Capital Medical University, Beijing 100015, China; 3 Department of Gastroenterology, Beijing Ditan Hospital, Capital Medical University, Beijing 100015, China; anwen1995@126.com (W.A.); weiherui123@163.com (H.W.); lmq452632622@163.com (M.L.); lindahell@163.com (L.H.); 4 Institute of Infectious Diseases, Beijing Ditan Hospital, Capital Medical University, Beijing 100015, China; xiaofan@ccmu.edu.cn

**Keywords:** pan-cancer analysis, *O*-GlcNAcylation, hepatocellular carcinoma, EOGT, angiogenesis, biomarker

## Abstract

**Background**: The EGF domain-specific O-GlcNAc transferase (EOGT), a migrasome marker, plays emerging roles in cancer biology through O-GlcNAcylation modifications, yet its pan-cancer functions and therapeutic implications remain underexplored. This study aimed to systematically characterize EOGT’s oncogenic mechanisms across malignancies, with particular focus on hepatocellular carcinoma (HCC) progression and sorafenib resistance. **Methods**: Multi-omics analysis integrated TCGA/GTEx data from 33 cancer types with spatial/single-cell transcriptomics and 10 HCC cohorts. Functional validation employed Huh7 cell models with *EOGT* modulation, RNA sequencing, and ceRNA network construction. Drug sensitivity analysis leveraged GDSC/CTRP/PRISM databases, while immune microenvironment assessment utilized ESTIMATE/TIMER algorithms. **Results**: *EOGT* showed cancer-specific dysregulation, marked by significant upregulation in HCC correlating with advanced stages and poor survival. Pan-cancer analysis revealed *EOGT*’s association with genomic instability, tumor stemness, and angiogenesis. Experimental validation demonstrated EOGT’s promotion of HCC proliferation and migration. A novel exosomal circ_0058189/miR-130a-3p/EOGT axis was identified, showing that circ_0058189 was upregulated in HCC tissues, plasma samples and exosomes of sorafenib-resistant cells. **Conclusion**: This study establishes EOGT as a pan-cancer angiogenesis biomarker, while elucidating its role in therapeutic resistance via exosomal circRNA-mediated regulation, providing mechanistic insights for targeted intervention strategies.

## 1. Introduction

*O*-GlcNAcylation, a dynamic post-translational modification characterized by the attachment of β-N-acetylglucosamine to serine/threonine residues of nuclear and cytoplasmic proteins, has emerged as a key regulator in cancer pathogenesis [1]. Recent studies have elucidated the functional consequences of this modification in various malignancies. In hepatocellular carcinoma (HCC), glucose-induced *O*-GlcNAcylation at Thr241 of YAP has been shown to modulate its transcriptional activity, promoting oncogenic transformation through enhanced protein stability and functionality [2]. Similarly, in colorectal cancer models, YY1 modification at Thr236 has been demonstrated to regulate downstream targets, including SLC22A15 and AANAT, establishing a molecular link between O-GlcNAcylation and tumor progression [3]. Pancreatic cancer studies have further revealed a metabolic connection where elevated folate metabolism drives increased *UDP*-GlcNAc production, subsequently enhancing cMYC modification and PD-L1 expression, thereby facilitating immune escape mechanisms [4].

While *O*-GlcNAc transferase (OGT) and *O*-GlcNAcase (OGA) are widely recognized as the primary enzymes regulating this modification cycle [5,6,7], emerging evidence suggests additional players in this process. Notably, EGF domain-specific *O*-GlcNAc transferase (EOGT) has been identified as a specialized enzyme mediating the *O*-GlcNAcylation of extracellular and membrane-associated proteins containing EGF-like repeats [8]. The biological significance of EOGT in cancer remains incompletely understood, though recent discoveries have begun to shed light on its potential roles. Intriguingly, EOGT has been identified as a biomarker of migrasomes—novel cellular structures implicated in intercellular communication through the transport of bioactive molecules, potentially influencing tumor cell motility and vascularization processes [9,10].

Molecular interactions involving EOGT are gaining attention in cancer research. In pancreatic cancer models, EOGT has been shown to form a complex with SHCBP1, leading to enhanced NOTCH1 modification and subsequent tumor progression [11]. Our initial investigations have further revealed a correlation between EOGT expression patterns and immune cell infiltration in HCC, suggesting its potential utility as a clinical prognostic indicator [12]. These collective findings underscore the need for deeper exploration of EOGT-mediated *O*-GlcNAcylation in cancer biology, particularly its role in tumor microenvironment regulation and therapeutic resistance mechanisms.

Although existing evidence suggests a possible regulatory role of EOGT across multiple malignancies, comprehensive investigations exploring its pan-cancer functions remain notably absent in current scientific literature. The predominant focus on individual cancer types in most studies may lead to an incomplete understanding of this target gene’s mechanistic significance, primarily due to the absence of a holistic analytical framework. This knowledge gap underscores the critical necessity for the systematic evaluation of *EOGT* expression patterns and their functional implications across diverse cancer subtypes, which would provide valuable insights for guiding subsequent translational research and therapeutic development.

In this investigation, we implemented a comprehensive poly-omics approach to elucidate EOGT’s pan-cancer implications through the systematic integration of multi-dimensional datasets encompassing diverse malignancies and corresponding normal tissues. Our pan-cancer analysis framework specifically focused on the following aspects: the expression patterns of *EOGT* and their clinical significance, the association between *EOGT* and genomic variations/genomic instability, the functional regulatory mechanisms of *EOGT* in DNA repair, tumor stemness, epigenetic regulation, alternative splicing, the signaling pathways mediated by EOGT, the interactions between EOGT and the tumor immune microenvironment, and the drug sensitivity analysis of EOGT. Through initially rigorous validation employing cellular models, we demonstrated EOGT’s functional impact on HCC progression. Furthermore, our results preliminarily revealed a potential new mechanism that exosome-derived circ_0058189 may be involved in HCC progression and sorafenib resistance through the miR-130a/EOGT axis.

## 2. Materials and Methods

### 2.1. Pan-Cancer Data Collection and Processing

*EOGT* expression data and clinical features from The Cancer Genome Atlas (TCGA) and Genotype-Tissue Expression (GTEx) databases were accessed via the University of California Santa Cruz (UCSC) Xena platform [13]. All 36 TCGA pan-cancer cohort abbreviations and their corresponding sample sizes are comprehensively listed in Table S1. Although the sample sizes for certain cancer types and their corresponding controls in the TCGA pan-cancer analysis were relatively limited, we integrated healthy tissue samples from the GTEx database for subsequent pan-cancer analyses. Ten HCC transcriptome datasets, including GSE102079, GSE102083, GSE121248, GSE136247, GSE45267, GSE45436, GSE62232, GSE64041, GSE76297, and GSE84005, were obtained from Gene Expression Omnibus (GEO), employing a multi-cohort strategy to mitigate potential biases inherent in single-study analyses. *EOGT* expression profiles across cancer cell lines were analyzed using Cancer Cell Line Encyclopedia (CCLE) [14], while Simple Nucleotide Variation (SNV) and methylation data were sourced from cBioPortal [15]. To ensure data robustness, inclusion criteria focused on 33 cancer types with matched normal tissues from these resources, prioritizing HCC datasets and EOGT-related functional pathways. Low-quality samples, non-cancer studies, and redundant entries were systematically excluded. Transcriptome data were normalized using log_2_ (Transcripts Per Million (TPM) + 1). EOGT protein sequences were retrieved from the National Center for Biotechnology Information (NCBI).

### 2.2. Pan-Cancer Analyses of Differential EOGT Expression

*EOGT* mRNA expression in tumors versus normal tissues across 33 cancers was compared using integrated TCGA and GTEx data. Differential expression was analyzed using R’s ggplot2 package. Protein-level differences were assessed via UALCAN [16], and expression variations by gender, stage, grade, and TNM classification were visualized.

### 2.3. Diagnostic and Prognostic Evaluation

Clinical data, including overall survival (OS), disease-specific survival (DSS), progression-free interval (PFI), and disease-free interval (DFI), were extracted from the TCGA and GTEx databases. OS was defined as the time from diagnosis to death from any cause, DSS as the time from diagnosis to death specifically from the disease, PFI as the time from diagnosis to disease progression or recurrence, and DFI as the time from the end of treatment to disease recurrence. Patients with incomplete or missing data were excluded from the analysis. Data normalization was performed using log2 transformation.

ROC curves were generated using the pROC package in R, with patients stratified by *EOGT* expression cutoffs determined by the Youden index. Kaplan–Meier survival curves were plotted to compare survival outcomes between high and low *EOGT* expression groups, and log-rank tests were used to assess statistical significance. Univariate Cox regression analysis was performed to evaluate the prognostic value of EOGT for OS, DSS, PFI, and DFI, with hazard ratios (HRs) and 95% confidence intervals (CIs) reported.

### 2.4. Genomic Alteration and Mutational Burden

cBioPortal’s Cancer Type Summary module [17] analyzed EOGT mutations, amplifications, and deletions. TCGA SNV data processed by MuTect2 [18] were integrated, and *EOGT* domain mutations were identified using R’s maftools [19]. Correlations between *EOGT* expression and tumor mutational burden (TMB), microsatellite instability (MSI), homologous recombination deficiency (HRD), neoantigens, tumor ploidy/aneuploidy, and silent mutation rate/nonsilent mutation rate were evaluated using Spearman’s correlation.

### 2.5. DNA Repair, Stemness, and Epigenetic Analysis

*EOGT*’s relationship with mismatch repair (MMR) [20] and homologous recombination repair (HRR) genes [21] was assessed using Gene Expression Profiling Interactive Analysis 2 (GEPIA2) [22]. Tumor stemness was evaluated via the differentially methylated probes-based stemness index (DMPsi) [23], and epigenetic modifications were analyzed for DNMTs [24] and RNA modification genes (N1-methyladenosine: m1A, 5-methylcytosine: m5C, N6-methyladenosine: m6A) [25]. EOGT methylation’s impact on survival was analyzed using cBioPortal data and R’s Survival package, and significance was set at *p* < 0.05.

### 2.6. Alternative Splicing Analysis

Clinically relevant *EOGT* alternative splicing (AS) events were identified using OncoSplicing’s ClinicalAS tool [26]. Percent spliced-in (PSI) values from SpliceSeq and SplAdder projects were compared between tumors and normal tissues using Student’s *t*-test or the Mann–Whitney U-test, with AS events in ≥3 cancers highlighted.

### 2.7. Protein Interaction and Functional Enrichment

EOGT’s protein interaction network was constructed using Search Tool for the Retrieval of Interacting Genes/Proteins (STRING) [27]. Reverse Phase Protein Array (RPPA) data from The Cancer Proteome Atlas (TCPA) assessed EOGT’s correlation with 10 cancer pathways [28]. Differential expression analysis (limma) and GSEA (ClusterProfiler) [29] identified enriched pathways visualized via bubble plots. Significant pathways were identified based on *p* < 0.05 and a false discovery rate (FDR) < 0.1.

### 2.8. Immune Response Analysis

ESTIMATE scores [30] quantified immune and stromal cell proportions in 33 cancers. EOGT expression differences across six immune subtypes (C1–C6) [31] were evaluated using TISIDB. Spearman’s correlation coefficients were calculated to assess the relationship between immune-related genes and checkpoint markers [32]. TISMO assessed *EOGT* expression changes post-cytokine and anti-PD-1 treatment [33]. Immune cell infiltration was quantified using EPIC, MCPcounter, CIBERSORT, and Xcell [34,35,36] and validated via TIMER2.0 [37]. Spatial and single-cell transcriptomic data (TISCH [19], CancerSEA [38]) further explored EOGT’s role in immune modulation.

### 2.9. Chemotherapeutic Drug Screening

EOGT’s association with drug sensitivity was explored using Cancer Therapeutics Response Portal (CTRP), Genomics of Drug Sensitivity in Cancer (GDSC), and Project on Precision Oncology for Every Nation (PRISM) databases. Samples were stratified by median EOGT expression, and drug sensitivity (AUC, IC50) was compared. Spearman correlations identified the top 30 drugs linked to EOGT expression, and the results were consolidated into a bubble heatmap for visualization. Connectivity Map (CMap) [39] identified potential EOGT inhibitors by matching gene signatures, with results summarized and visualized.

### 2.10. Cell Line, Lentivirus Infection, and Transfection of miRNA Mimics

The original cell line was obtained from the Cell Resource Center of the Chinese Academy of Medical Sciences (CAMS). Hepatoma cell line Huh7 was cultured with Dulbecco’s modified Eagle’s medium, High Glucose (EallBio, Beijing, China) containing 10% fetal bovine serum (FBS, ExCell Bio, Suzhou, China), 100 U/mL penicillin, and 100 μg/mL streptomycin. Cells were cultured in 5% CO_2_ and 37 °C conditions.

To induce *EOGT* overexpression in hepatoma cells, a lentiviral vector system was utilized, specifically the pCDH-CMV-MCS-EF1-CopGFP-T2A-Puro construct (TranSheepBio, Shanghai, China). Huh7 cells, a hepatoma cell line, were seeded into six-well plates and maintained in DMEM supplemented with 10% FBS and 1% penicillin/streptomycin. When cell confluence reached 30–40%, lentiviral particles carrying the EOGT gene (NM_001278689.1) were introduced at a multiplicity of infection (MOI) of 20. Following a 72 h incubation, puromycin selection was initiated at 2 µg/mL and maintained for 3–7 days, with the culture medium refreshed every 1–2 days to ensure optimal cell growth and selection efficiency. For *EOGT* knockdown experiments, hepatoma cells were transduced with lentiviral plasmids (LV3-H1/GFP&Puro, GenePharma, Suzhou, China) designed to express *EOGT*-specific short hairpin RNA (shRNA) or a control shRNA. The shRNA target sequence used was GCGAACCTCTGTATAACTATG (5′ to 3′). Incubation and screening processes were performed as before.

Transfections of miRNA mimics were performed using Lipofectamine 3000 (Thermo Fisher Scientific, Waltham, MA, USA) in Opti-MEM medium (Thermo Fisher Scientific, Waltham, MA, USA). All the miRNA mimics were purchased from GenePharma (Suzhou, China), and the sequence information of the miRNA mimics used for transient transfection is shown in Table S2.

### 2.11. Western Blot Analysis

Radioimmunoprecipitation Assay (RIPA, Beyotime, Nanjing, China) buffer was used to extract total protein lysates from cells or tissues. The protein lysates were subjected to SDS-PAGE and then transferred to a nitrocellulose membrane. The membrane was blocked with 5% non-fat milk for 1 h and incubated with the first antibody for >12 h at 4 °C. After incubation with the secondary antibody for 1 h at 37 °C, the membrane was visualized by ECL Western Blotting Substrate from Thermo Fisher Scientific (Cleveland, OH, USA). All the antibodies are listed in Supplementary Table S3.

### 2.12. RNA-Seq and Bioinformatic Analysis

Total RNA was isolated from two distinct groups of Huh7 cells: those overexpressing *EOGT* (*n* = 3) and those with *EOGT* knockdown (*n* = 3). Subsequently, RNA-seq analysis was performed on the isolated RNA by Gene-Denovo (Guangzhou, China). Data analysis was carried out utilizing the online platform OmicShare, along with Sangerbox [40] and STRING [41]. Additional bioinformatics resources that were employed comprised the GEPIA2 [22], Biomarker Exploration for Solid Tumors (BEST) [42], the Integrative HCC Gene Analysis (IHGA) [43], and the UALCAN database [16]. The spatial transcriptome data came from the study of Liu et al. [44].

### 2.13. ceRNA Network Construction and Validation

To construct the EOGT-centered ceRNA network, candidate miRNAs targeting *EOGT* were screened using mirDIP (score class: High; top 5% ranking; Integrated Score > 0.7) and miRwalk (stringency score = 1; Targetscan, Mirdb, Mirtarbase databases), followed by intersection with downregulated miRNAs (log2FC ≥ 1, adjusted *p* < 0.01) identified from the CancerMIRNome database. The prognostic validation of miRNAs was performed using the dbDEMC database and survival analysis of HCC patient data. Exosomal circRNAs were analyzed using the GEO dataset GSE101850 (sorafenib-resistant vs. sensitive HepG2 cells; *p* <0.05), and circRNA-miRNA interactions were predicted via circMINE. Functional validation included Western blotting for miRNA-mediated *EOGT* regulation. The external validation of circRNAs utilized GEO datasets GSE166678 (HCC plasma) and GSE97332 (HCC tissues), with host gene expression and survival analysis conducted using TCGA data. Binding interactions were confirmed via Targetscan (miRNA-*EOGT*) and circBase (circRNA-miRNA).

### 2.14. Statistical Analysis

All analyses and graphs were generated with R_4.2.1 and GraphPad Prism 8. Statistical significance was assessed by comparing mean values (±SD) using a Student’s *t*-test for independent groups when the assumptions of normality (Kolmogorov–Smirnov test) and homogeneity of variance (Bartlett’s test) were met. For datasets that violated these assumptions, Welch’s *t*-test or the Mann–Whitney U-test was used. Significance was expressed for * *p* < 0.05; ** *p* < 0.01; *** *p* < 0.001; **** *p* < 0.0001.

Survival comparisons were performed using the Kaplan–Meier method, and statistical significance was assessed using the log-rank test. Univariate Cox regression analysis was conducted to evaluate the prognostic value of *EOGT* expression, with hazard ratios (HRs) and 95% confidence intervals (CIs) reported. For correlation analyses, Spearman’s rank correlation coefficient was used to assess the relationship between variables.

## 3. Results

### 3.1. Pan-Cancer Landscape of EOGT Expression and Clinical Relevance

**Figure 1:**
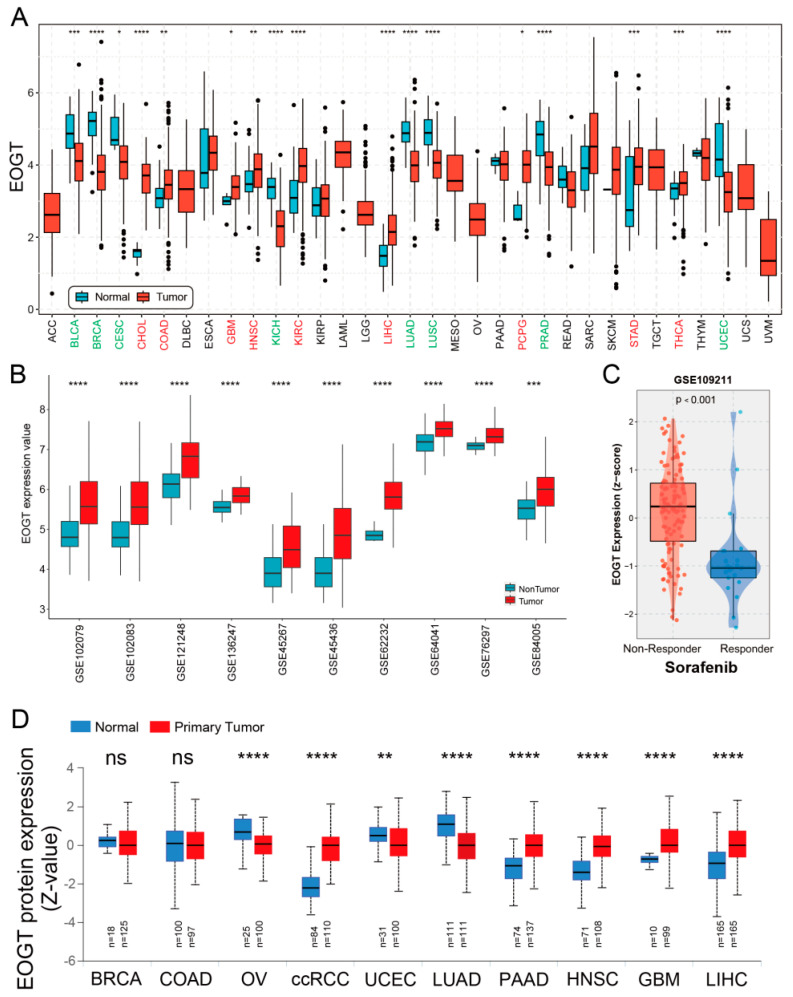
Pan-cancer analysis of *EOGT* expression. (**A**) Systematic pan-cancer analysis of *EOGT* mRNA expression using TCGA and GTEx databases showing differential expression across over 30 cancer types. (**B**) *EOGT* expression in 10 HCC transcriptome datasets, indicating significantly higher expression in HCC tissues compared to adjacent normal or healthy tissues. (**C**) Significant upregulation of *EOGT* in tumor tissue samples from sorafenib non-responders. (**D**) UALCAN data presenting protein-level differences in EOGT in various cancers. Upregulated EOGT protein levels were observed in KIRC, PAAD, HNSC, GBM, and LIHC, while downregulation was seen in OV, UCEC, and LUAD, which is consistent with transcriptomic findings. ccRCC, clear cell renal cell carcinoma; full terminology for abbreviations is provided in Table S1; * *p* < 0.05, ** *p* < 0.01, *** *p* < 0.001, **** *p* < 0.0001, ns, not significant.

A systematic pan-cancer analysis of *EOGT* mRNA expression was conducted using TCGA and GTEx databases, revealing differential expression across 33 cancer types (Figure 1A). Notably, *EOGT* was significantly upregulated in CHOL, COAD, GBM, HNSC, KIRC, LIHC, PCPG, STAD, and THCA (*p* < 0.05, Student’s *t*-test), while downregulated in BLCA, BRCA, CESC, KICH, LUAD, LUSC, PRAD, and UCEC compared to normal tissues (*p* < 0.05). In 10 HCC transcriptome datasets, *EOGT* expression was significantly higher in HCC tissues compared to adjacent normal or healthy tissues (Figure 1B). Notably, analysis of GSE109211 data from the BEST database demonstrated significant upregulation of *EOGT* in tumor tissue samples from sorafenib non-responders (Figure 1C). Relative *EOGT* expression levels across various cell lines obtained from CCLE are shown in Figure S1A. UALCAN data indicated upregulated EOGT protein levels in KIRC, PAAD, HNSC, GBM, and LIHC, while downregulation was observed in OV, UCEC, and LUAD, consistent with transcriptomic findings (Figure 1D). We further evaluated *EOGT* expression across 33 tumor types, stratifying the analysis by gender, clinical stage, tumor grade, and TNM classification (Figure S1B). Notably, *EOGT* expression showed a significant positive correlation with advanced tumor grades in HNSC, STAD, GBMLGG, and LGG, suggesting its potential role in promoting tumor progression. Additionally, in tumors such as KIPAN, SKCM, and ACC, *EOGT* expression was positively associated with distant metastasis, further supporting its involvement in aggressive cancer phenotypes. In our focused analysis of HCC, *EOGT* expression exhibited a strong positive correlation with both clinical stage and tumor grade. Statistically significant differences were observed in comparisons between grade 1 vs. 2 (*p* = 2.01 × 10^−2^), grade 1 vs. 3 (*p* = 4.41 × 10^−4^), and stage 1 vs. 3 (*p* = 1.45 × 10^−2^). These findings highlight EOGT as a potential biomarker for HCC progression and aggressiveness.

### 3.2. Diagnostic and Prognostic Value of EOGT in Pan-Cancers

**Figure 2:**
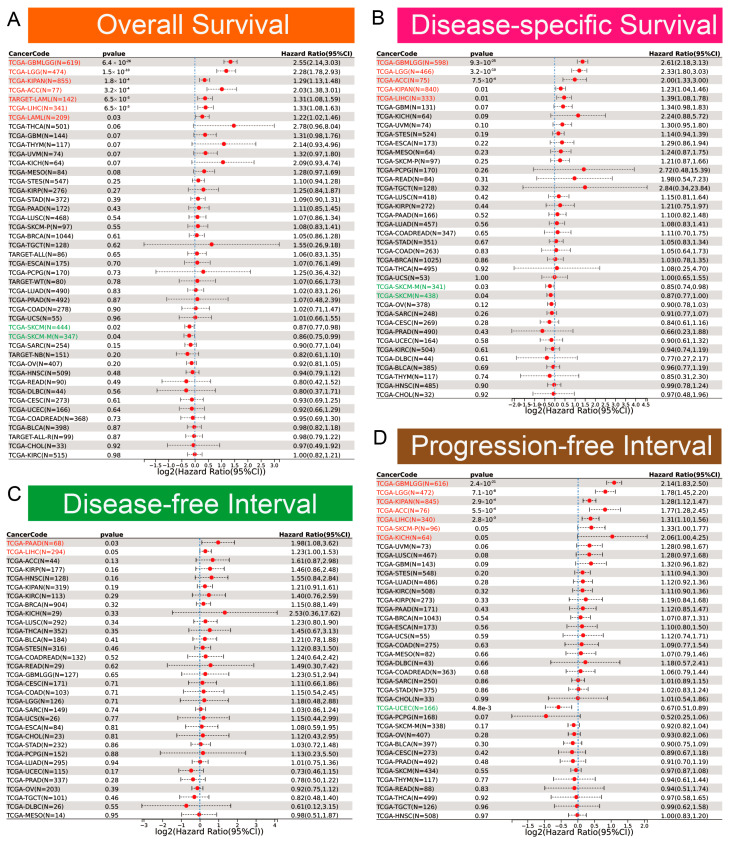
Diagnostic and prognostic value of *EOGT* in multiple cancers. (**A**–**D**): Univariate Cox regression analysis demonstrating the association between high *EOGT* expression and overall survival (OS), disease-specific survival (DSS), disease-free interval (DFI), and progression-free interval (PFI). Full terminology for abbreviations is provided in Table S1.

Clinically, *EOGT* served as a robust prognostic marker. ROC curve analysis revealed *EOGT*’s potential as a diagnostic biomarker across multiple cancer types, with area under the curve (AUC) values exceeding 0.8 in over 11 malignancies. Notably, in LIHC, EOGT demonstrated high diagnostic accuracy (AUC = 0.844) (Figure S2). Univariate Cox regression analysis revealed that high *EOGT* expression was associated with poorer OS in GBMLGG, LGG, KIPAN, ACC, LAML, and LIHC (log-rank test, *p* < 0.05) while acting as a protective factor in SKCM (*p* < 0.05, Figure 2A). Similarly, *EOGT* was a risk factor for worse DSS in GBMLGG, LGG, KIPAN, ACC, and LIHC but protective in SKCM (*p* < 0.05, Figure 2B). For DFI, *EOGT* was a risk factor in PAAD and LIHC (*p* < 0.05, Figure 2C), while for PFI, it was a risk factor in GBMLGG, LGG, KIPAN, ACC, LIHC, SKCM, and KICH but protective in UCEC (*p* < 0.05, Figure 2D). Kaplan–Meier curves further validated these findings (Figure S3), indicating that high *EOGT* expression generally correlates with poor prognosis in LGG, KIPAN, ACC, LAML, and LIHC, with LIHC consistently identified as a high-risk cancer across all survival metrics (log-rank test, *p* < 0.05).

### 3.3. Genomic Alterations and Instability of EOGT

**Figure 3:**
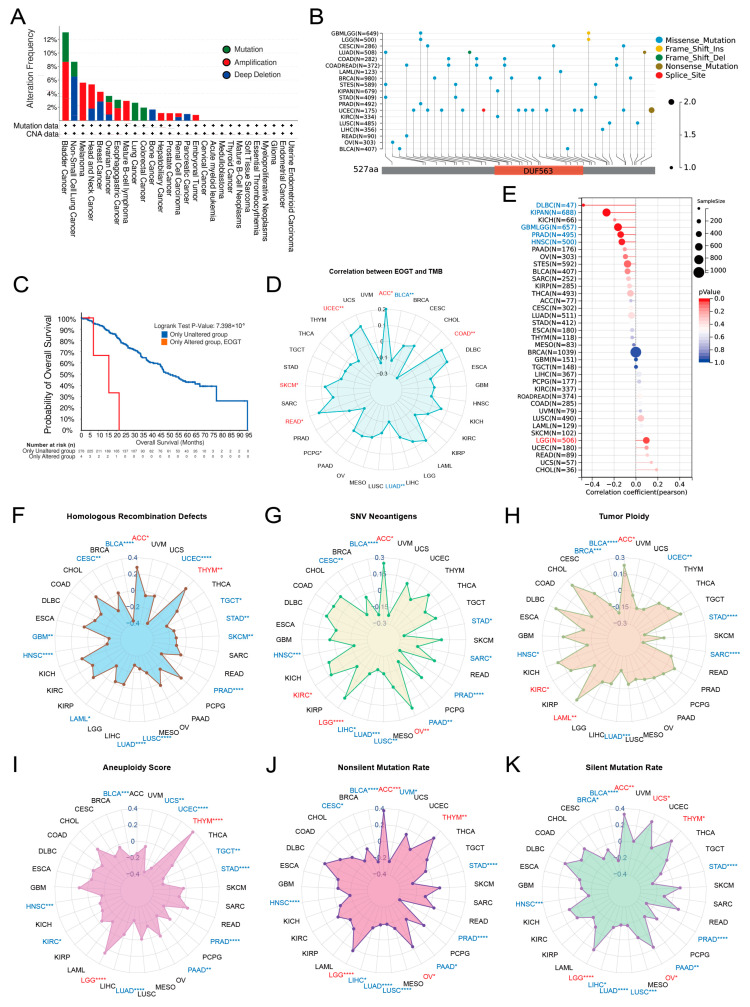
Genomic alterations and instability of *EOGT*. (**A**,**B**) Pan-cancer analysis of *EOGT* copy number variations (CNVs) and single-nucleotide variations (SNVs) using cBioPortal and GDC databases. (**C**) Survival analysis showing significantly lower survival rates in patients with *EOGT* mutations (*p* < 0.01). (**D**) Correlations between *EOGT* expression and tumor mutational burden (TMB). (**E**) Correlations between *EOGT* expression and microsatellite instability (MSI). (**F**) Correlations between *EOGT* expression and homologous recombination deficiency (HRD). (**G**) Correlations between *EOGT* expression and single-nucleotide variant (SNV) neoantigens. (**H**,**I**) Analyses of correlations between *EOGT* expression and tumor ploidy and aneuploidy. (**J**) Correlations between *EOGT* expression and non-silent mutation rates. (**K**) Correlations between *EOGT* expression and silent mutation rates. Full terminology for abbreviations is provided in Table S1; * *p* < 0.05, ** *p* < 0.01, *** *p* < 0.001, **** *p* < 0.0001.

Pan-cancer analysis of *EOGT* copy number variations (CNVs) and SNVs using cBioPortal and GDC databases revealed gene amplifications in bladder cancer, melanoma, head and neck cancer, esophageal cancer, diffuse large B-cell lymphoma, hepatobiliary tumors, prostate cancer, and germ cell tumors, while deep deletions were common in non-small cell lung cancer, breast cancer, bone tumors, and pancreatic cancer. High SNV rates were observed in endometrial, intestinal, and breast cancers (Figure 3A,B). Survival analysis showed significantly lower survival rates in patients with EOGT mutations (*p* < 0.01, Figure 3C).

Correlations between *EOGT* expression and genomic instability markers, including TMB, MSI, HRD, SNV neoantigens, tumor ploidy, aneuploidy, and mutation rates, were evaluated [45,46]. Positive correlations with TMB were observed in ACC, COAD, READ, SKCM, and UCEC, while negative correlations were noted in BLCA and LUAD (Figure 3D). *EOGT* positively correlated with MSI in LGG but negatively in DLBC, KIPAN, GBMLGG, PRAD, and HNSC (Figure 3E). Positive correlations with HRD were found in ACC and THYM, while negative correlations were observed in BLCA, CESC, GBM, HNSC, LAML, LUAD, LUSC, PRAD, SKCM, STAD, TGCT, and UCEC (Figure 3F). *EOGT* also showed positive correlations with SNV neoantigens in ACC, KIRC, LGG, and OV but negative correlations in BLCA, CESC, HNSC, LIHC, LUAD, LUSC, PAAD, PRAD, SARC, and STAD (Figure 3G). Tumor ploidy analyses revealed positive correlations in ACC, KIRC, and LAML but negative correlations in BLCA, BRCA, HNSC, LUAD, SARC, STAD, and UCEC (Figure 3H). Tumor aneuploidy analyses revealed positive correlations in THYM and LGG but negative correlations in BLCA, HNSC, KIRC, LUAD, PAAD, PRAD, STAD, TGCT, UCEC, and UCS (Figure 3I). *EOGT* positively correlated with nonsilent mutation rates in ACC, LGG, OV, and THYM but negatively in BLCA, CESC, HNSC, LIHC, LUAD, LUSC, PAAD, PRAD, and STAD (Figure 3J). Similarly, positive correlations with silent mutation rates were observed in ACC, LGG, OV, THYM, and UCS, while negative correlations were noted in BLCA, HNSC, LIHC, LUAD, LUSC, PAAD, PRAD, and STAD (Figure 3K). These findings suggest a significant association between *EOGT* and genomic instability, highlighting its potential as a target for immunotherapy.

### 3.4. EOGT Expression and DNA Repair, Stemness, and Epigenetic Modifications

**Figure 4:**
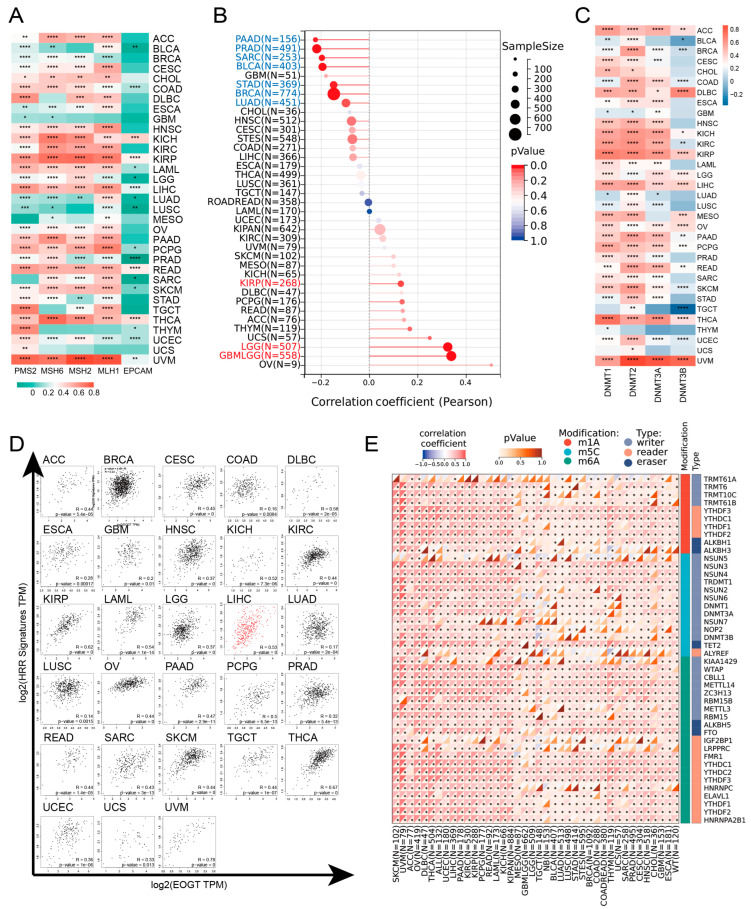
*EOGT* expression and DNA repair, stemness, and epigenetic modifications. (**A**) Correlations between *EOGT* expression and mismatch repair (MMR) and homologous recombination repair (HRR) genes. (**B**) Correlations between *EOGT* and tumor stemness. (**C**) Correlations between *EOGT* and DNA methyltransferases (DNMTs). (**D**) Positive correlations with HRR gene signatures in 28 cancers. (**E**) Significant correlations between *EOGT* and RNA modification genes. Full terminology for abbreviations is provided in Table S1, * *p* < 0.05, ** *p* < 0.01, *** *p* < 0.001, **** *p* < 0.0001.

*EOGT* expression was positively correlated with MMR and HRR genes in most cancers, except GBM, MESO, THYM, and UCS, with particularly strong correlations in KICH, KIRP, LIHC, READ, THCA, and UVM (Figure 4A). Positive correlations with HRR gene signatures were also observed in 28 cancers, including ACC, LIHC, SKCM, and THCA (Figure 4D). Additionally, *EOGT* positively correlated with tumor stemness in KIRP, LGG, and GBMLGG (Figure 4B), suggesting its role in regulating DNA damage repair and cancer progression.

Epigenetic analysis revealed significant positive correlations between *EOGT* and DNMTs in ACC, DLBC, KICH, KIRP, LGG, LIHC, OV, PAAD, THCA, and UVM (Figure 4C). *EOGT* expression was inversely correlated with promoter methylation in 30 cancer types (Figure S4), and reduced methylation predicted shorter survival in LGG and THYM (Figure S5). *EOGT* also showed significant correlations with RNA modification genes (Figure 4E), indicating its potential role in DNA methylation and mRNA regulation.

### 3.5. EOGT Alternative Splicing and Survival Outcomes

**Figure 5:**
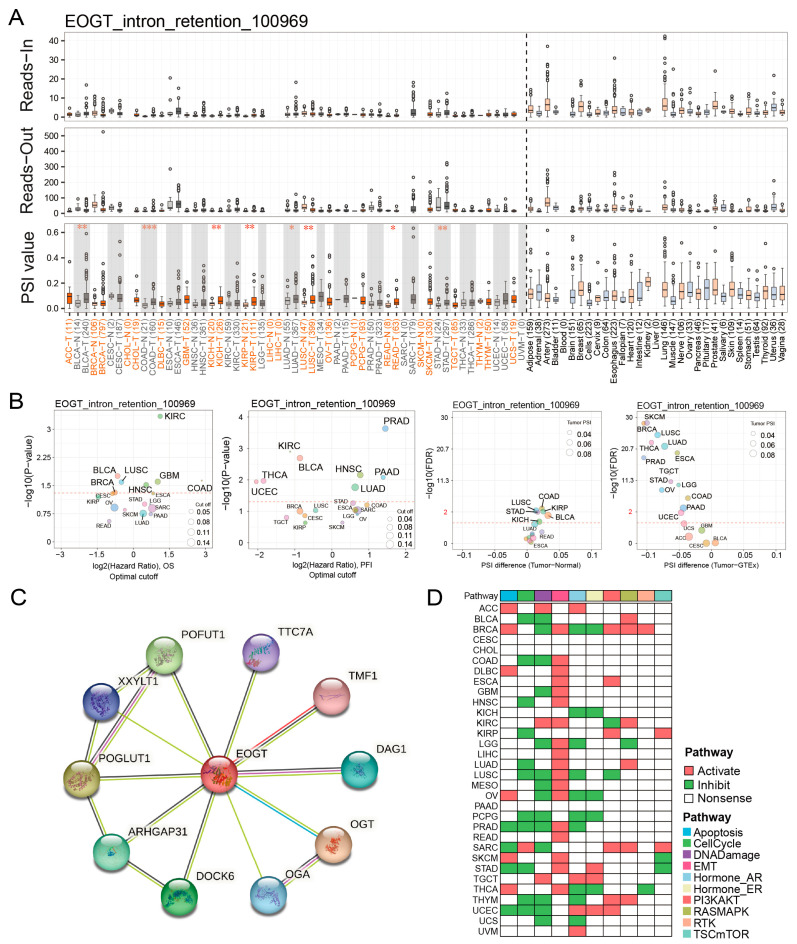
*EOGT* alternative splicing and survival outcomes. (**A**,**B**) Fifteen clinically relevant *EOGT* alternative splicing (AS) events were identified using OncoSplicing. The EOGT_intron_retention_100969 event, present in ≥3 cancer types, showed higher percent spliced-in (PSI) values in BLCA, COAD, KICH, KIRP, LUAD, LUSC, READ, and STAD compared to normal tissues, and optimal PSI cut-off-based analysis showed significant OS and PFI differences across tumors, the red dashed line denotes the *p*-value threshold (*p* = 0.05). (**C**) STRING analysis identifying 10 potential EOGT-interacting proteins. (**D**) Pathway enrichment analysis using TCPA data, revealing that high EOGT expression was associated with activated epithelial-to-mesenchymal transition (EMT) and suppressed DNA damage response pathways. Full terminology for abbreviations is provided in Table S1; * *p* < 0.05, ** *p* < 0.01, *** *p* < 0.001.

Fifteen clinically relevant *EOGT* alternative splicing (AS) events were identified using OncoSplicing (Table S4). The EOGT_intron_retention_100969 event, present in ≥3 cancer types, showed higher PSI values in BLCA, COAD, KICH, KIRP, LUAD, LUSC, READ, and STAD compared to normal tissues, prognostic analysis based on the optimal PSI cut-off value revealed significant differences in OS and PFI across multiple tumor types (Figure 5A,B). These findings suggest that *EOGT* AS events may significantly impact cancer progression.

### 3.6. EOGT Promotes Cancer via EMT and DNA Damage Suppression

STRING analysis identified 10 potential EOGT-interacting proteins, indicating unexplored mechanisms (Figure 5C). Pathway enrichment analysis using TCPA data revealed that high EOGT expression was associated with activated EMT and suppressed DNA damage response pathways (Figure 5D). GSEA further linked high EOGT expression to the activation of pro-cancer pathways, including EMT, angiogenesis, inflammatory response, and TNFα signaling via NFκB, while suppressing oxidative phosphorylation (Figure S6), suggesting EOGT’s involvement in multiple oncogenic pathways.

### 3.7. EOGT and Immune Cell Infiltration in Tumors

**Figure 6:**
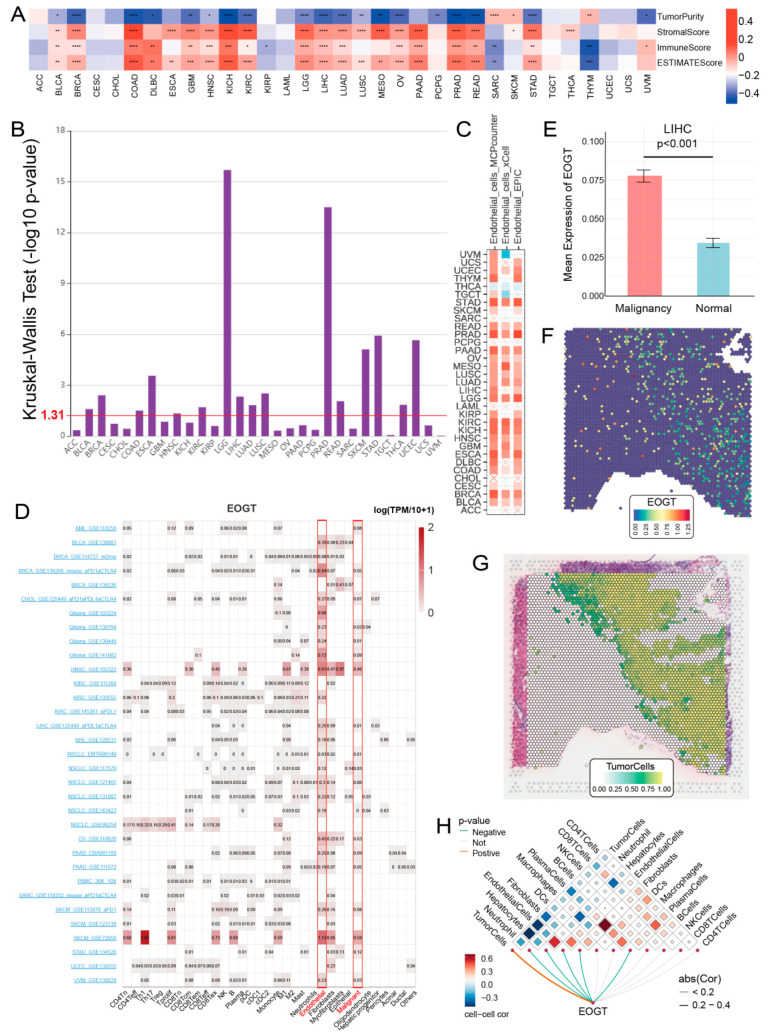
*EOGT* and immune cell infiltration in tumors. (**A**) The ESTIMATE algorithm was used to evaluate the association between *EOGT* and immunological characteristics across 33 cancer types. A significant positive correlation between ESTIMATE scores and *EOGT* expression levels was observed in 19 cancer types. (**B**) Analysis using the TISIDB database showing significant differences in *EOGT* expression across immunological subtypes in 16 cancer types. *EOGT* exhibited upregulation in the C6 immunological subtype of BRCA, COAD, HNSC, KIRC, LUAD, LUSC, STAD, and THCA, suggesting a potential functional relationship with TGF-β signaling activity. (**C**) TIMER2.0 analysis showing that *EOGT* expression positively correlated with endothelial cell abundance in multiple cancers. (**D**) TISCH single-cell data confirmed *EOGT* upregulation in endothelial and malignant cells, highlighted by red boxes across pan-cancer datasets. (**E**–**H**) Spatial transcriptomics revealing overlapping EOGT expression patterns with tumor cells in LIHC. Full terminology for abbreviations is provided in Table S1; * *p* < 0.05, ** *p* < 0.01, *** *p* < 0.001, **** *p* < 0.0001.

To elucidate the role of EOGT in the tumor immune microenvironment (TIME), we employed the ESTIMATE algorithm to assess its association with immunological characteristics across 33 cancer types. Strikingly, *EOGT* expression showed a significant positive correlation with ESTIMATE scores in 19 cancer types, underscoring its potential involvement in immune modulation. Conversely, negative correlations were observed in SARC and THYM (Figure 6A). Scatter plots highlighting the strongest correlations in the top six cancer types further illustrate these relationships (Figure S7A).

Building on these findings, we leveraged the TISIDB database to investigate *EOGT* expression across immunological subtypes. Significant differences were identified in 16 cancer types, with *EOGT* particularly upregulated in the C6 immunological subtype of BRCA, COAD, HNSC, KIRC, LUAD, LUSC, STAD, and THCA (Figure 6B). This upregulation suggests a potential functional link between *EOGT* and TGF-β signaling activity, a pathway known to play a critical role in immune evasion and tumor progression (Figure S7B).

To further explore EOGT’s role in immune regulation, we conducted a pan-cancer analysis of its correlations with immune-related genes (Figure S8). Additionally, using the TISMO database, we compared *EOGT* expression in samples before and after treatment with immune checkpoint inhibitors (ICIs) in vivo (Figure S9A) and in tumor cell lines treated with cytokines in vitro (Figure S9B). Notably, *EOGT* expression was upregulated in the TNFα-treated mouse breast cancer cell line 4T1. The effects of IFNβ and IFNγ on *EOGT* expression varied across cell lines, with IFNγ generally inducing upregulation and IFNβ more frequently causing downregulation. In ICI-treated samples, *EOGT* expression levels were significantly reduced in responder groups in some datasets, while no significant changes were observed in non-responder groups across all datasets.

Collectively, these findings reveal the complex and multifaceted relationship between *EOGT* expression and immune cell infiltration. *EOGT*’s modulation by cytokines and its differential expression in response to ICIs highlight its potential as a key player in tumor immunology. These insights pave the way for future investigations into EOGT as a biomarker for immune response and a therapeutic target in cancer immunotherapy.

### 3.8. EOGT as a Biomarker for Tumor Angiogenesis

To better understand the association between *EOGT* expression and cancer-related immune activity, we assessed the correlation between *EOGT* expression and the infiltration of various immune cell types (Figure S10). The TIMER2.0 analysis shows that *EOGT* expression positively correlated with endothelial cell abundance in multiple cancers (Figure 6C). Single-cell transcriptomic data from TISCH confirmed *EOGT* upregulation in endothelial and malignant cells (Figure 6D). Spatial transcriptomics revealed overlapping *EOGT* expression patterns with tumor cells in LIHC (Figure 6E–H), SKCM, and GBM (Figure S11A–H), suggesting its promotional role in tumors. CancerSEA analysis linked *EOGT* to angiogenesis, cell differentiation, EMT, inflammation, metastasis, and stemness while showing weak or negative correlations with cell cycle, DNA repair, and DNA damage (Figure S11I,J).

### 3.9. EOGT and Chemotherapy Sensitivity

Through a comprehensive analysis of the GDSC1/2, CTRP, and PRISM databases, we identified a significant association between high EOGT expression and resistance to multiple chemotherapeutic agents. Drug sensitivity was evaluated using two key metrics: IC50 (half-maximal inhibitory concentration; higher IC50 indicates stronger resistance) and AUC (area under the concentration-effect curve; higher AUC suggests greater drug sensitivity). In GDSC1/2, IC50 was used, while CTRP and PRISM employed AUC. As illustrated in Figure S12A, EOGT shows a clear correlation with resistance to various drugs, particularly tyrosine kinase inhibitors (TKIs). For example, Afatinib demonstrated correlations of 0.246 (*p* = 1.65 × 10^−10^) in CTRP and 0.215 (*p* = 2.40 × 10^−6^) in PRISM. Similarly, Lapatinib exhibited correlations of 0.277 (*p* = 4.49 × 10^−13^) in CTRP, −0.333 (*p* < 0.001) in GDSC1, and 0.215 (*p* = 2.32 × 10^−6^) in PRISM. Gefitinib showed correlations of 0.120 (*p* = 0.00203) in CTRP and 0.183 (*p* = 6.39 × 10^−5^) in PRISM, while Selumetinib displayed correlations of −0.179 (*p* = 2.80 × 10^−7^) in GDSC1 and −0.226 (*p* = 3.16 × 10^−9^) in GDSC2.

To identify potential EOGT inhibitors, we utilized the Connectivity Map (CMap) database, which compares disease-associated gene expression profiles with drug-induced gene expression changes using pattern-matching algorithms. The core hypothesis is that if a drug-induced expression profile reverses the disease-associated expression pattern, the drug may have therapeutic potential. In the context of EOGT, negative correlation scores indicate that the drug may inhibit EOGT-related pro-tumorigenic pathways, with lower scores suggesting stronger inhibition. As shown in the heatmap of Figure S12B, we integrated the three lowest-scoring (i.e., most inhibitory) drugs for each cancer type. Statistical analysis revealed that arachidonyltrifluoromethane and W.13 each appeared nine times (across nine cancer types, including ACC, BRCA, and KICH), while MK-886 and X4.5.dianilinophthalimide each appeared eight times (involving eight cancer types, such as BLCA, GBM, and MESO). The significant enrichment of these four drugs across multiple cancer types suggests their potential to target EOGT or its synergistic pathways, providing promising candidates for the development of EOGT-targeted therapies.

### 3.10. EOGT Facilitates HCC Cell Proliferation and Migration

**Figure 7:**
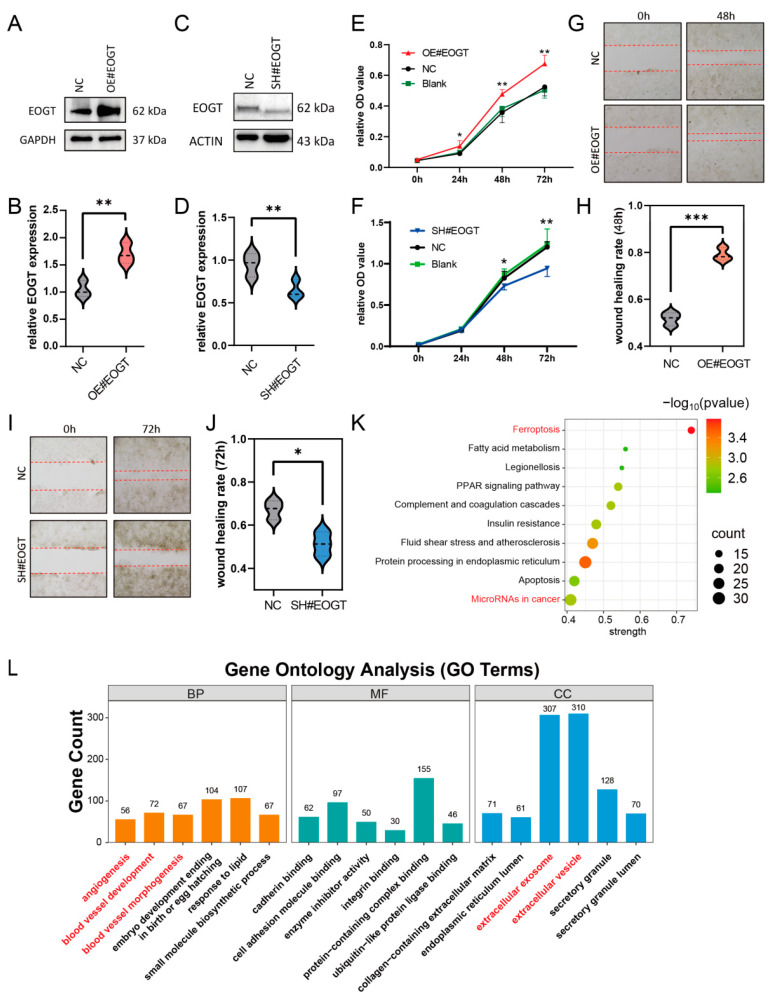
EOGT facilitates HCC cell proliferation and migration. (**A**–**D**) Stable knockdown and overexpression of *EOGT* in the Huh7 cell line were achieved, as confirmed by Western blot analyses. (**E**,**F**) The CCK8 assay demonstrates that Huh7-OE#EOGT and Huh7-SH#EOGT cells exhibited elevated and reduced proliferation compared to control cells, respectively. (**G**–**J**) The wound healing assay indicates that overexpression of *EOGT* promoted cell migration, while knockdown of *EOGT* had the opposite effect. (**K**,**L**) KEGG pathway and gene ontology (GO) analysis of RNA-seq results from Huh7 cells with *EOGT* overexpression and knockdown, showing significant enrichment in several cancer-related pathways and biological processes, including microRNA regulation in cancer, angiogenesis, etc.; * *p* < 0.05, ** *p* < 0.01, *** *p* < 0.001.

To investigate the role of EOGT in cellular behavior, the stable knockdown and overexpression of *EOGT* were achieved in the Huh7 cell line (Figure 7A–D). The CCK8 assay demonstrated that Huh7-OE#EOGT and Huh7-SH#EOGT cells exhibited elevated and reduced proliferation compared to control cells, respectively (Figure 7E,F). Additionally, the wound healing assay indicated that overexpression of *EOGT* promotes cell migration, while the knockdown of *EOGT* was just the opposite (Figure 7G–J).

To elucidate the functional consequences of EOGT modulation in HCC, we established isogenic Huh7 cell models with stable *EOGT* knockdown and overexpression, as confirmed by Western blot analyses (Figure 7A–D). Functional characterization revealed that *EOGT* expression levels significantly influenced cellular proliferation kinetics, with *EOGT*-overexpressing clones (Huh7-OE#EOGT) demonstrating enhanced growth potential, while *EOGT*-depleted cells (Huh7-SH#EOGT) showed attenuated proliferation rates in CCK-8 assays (Figure 7E,F). Furthermore, the quantitative assessment of cell motility through wound healing assays demonstrated a positive correlation between *EOGT* expression levels and migratory capacity, with *EOGT*-overexpressing cells displaying accelerated wound closure compared to their controls (Figure 7G–J).

To gain deeper insights into the molecular mechanisms underlying EOGT-mediated oncogenic effects, we performed transcriptomic profiling of *EOGT*-modulated Huh7 cells (*n* = 3 per group). Comparative RNA-seq analysis identified 1325 differentially expressed genes (DEGs) with significant expression changes (fold change ≥ 2, FDR < 0.01), comprising 426 upregulated and 879 downregulated transcripts associated with *EOGT* overexpression. Pathway enrichment analysis using the STRING database identified several EOGT-associated oncogenic pathways, particularly highlighting its involvement in cancer-related microRNA networks, vascular development processes (angiogenesis, blood vessel morphogenesis), and extracellular vesicle-mediated communication (Figure 7K,L). These findings collectively suggest that EOGT exerts multifaceted effects on HCC progression through the regulation of diverse oncogenic pathways.

### 3.11. EOGT-Mediated ceRNA Network in HCC Progression and Sorafenib Resistance

Given the pivotal role of EOGT in HCC progression and sorafenib resistance, combined with the significant enrichment of exosome-related pathways and microRNA functions in our GO and KEGG analyses, we constructed a competitive endogenous RNA (ceRNA) network centered on *EOGT*. This network explores potential exosomal circRNA/microRNA/*EOGT* regulatory axes that may drive HCC progression and therapeutic resistance.

To identify *EOGT*-targeting miRNAs, we performed integrated screening using mirDIP and miRwalk databases under stringent criteria. In mirDIP, we set the score class to High, selected the top 5% ranking miRNAs, and applied an Integrated Score threshold >0.7, yielding 20 candidate miRNAs (Table S5). For miRwalk, we used the maximum stringency (Score = 1) and analyzed three genomic regions (coding sequences, 3′-UTR, and 5′-UTR) using Targetscan, Mirdb, and Mirtarbase databases, which identified 67 additional miRNAs. The combined dataset from both databases comprised 79 unique miRNAs (Table S6).

**Figure 8:**
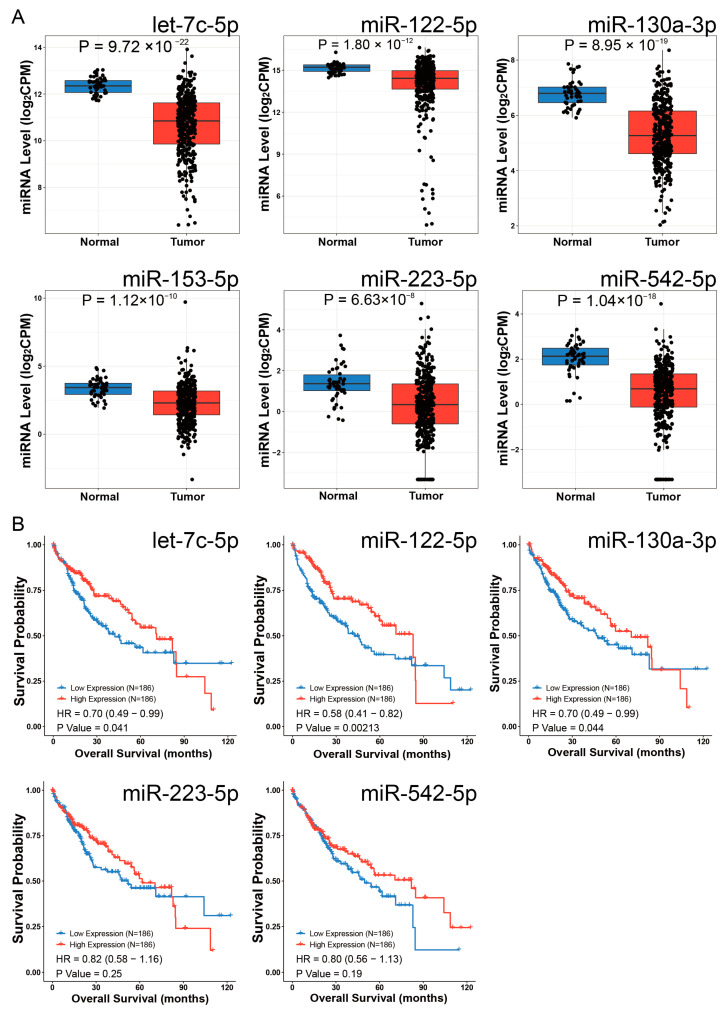
Identification of potential *EOGT*-targeting miRNAs. (**A**) Differential expression analysis using the CancerMIRNome database identified 109 downregulated miRNAs in HCC. Intersection with the initial 79 miRNAs from mirDIP and miRwalk databases revealed six potential *EOGT*-targeting miRNAs: hsa-let-7c-5p, hsa-miR-122-5p, hsa-miR-130a-3p, hsa-miR-153-5p, hsa-miR-223-5p, and hsa-miR-542-5p. (**B**) Analysis of the prognostic significance of the five miRNAs (after excluding hsa-miR-153-5p) showing that high expression of hsa-let-7c-5p, hsa-miR-122-5p, and hsa-miR-130a-3p was significantly associated with improved OS in HCC patients.

Differential expression analysis using the CancerMIRNome database identified 109 downregulated miRNAs in HCC (log2FC = 1, adjusted *p* < 0.01). Intersection with our initial 79 miRNAs revealed six potential *EOGT*-targeting miRNAs: hsa-let-7c-5p, hsa-miR-122-5p, hsa-miR-130a-3p, hsa-miR-153-5p, hsa-miR-223-5p, and hsa-miR-542-5p (Figure 8A). To validate the robustness of their downregulation in HCC, we queried the dbDEMC database, which confirmed the downregulation of all except hsa-miR-153-5p, reducing our candidate list to five miRNAs (Table 1). Further analysis of the prognostic significance of these five miRNAs revealed that the high expression of hsa-let-7c-5p, hsa-miR-122-5p, and hsa-miR-130a-3p was significantly associated with improved OS in HCC patients (Figure 8B). These three miRNAs were, therefore, selected as the core components for constructing the ceRNA network.

**Figure 9:**
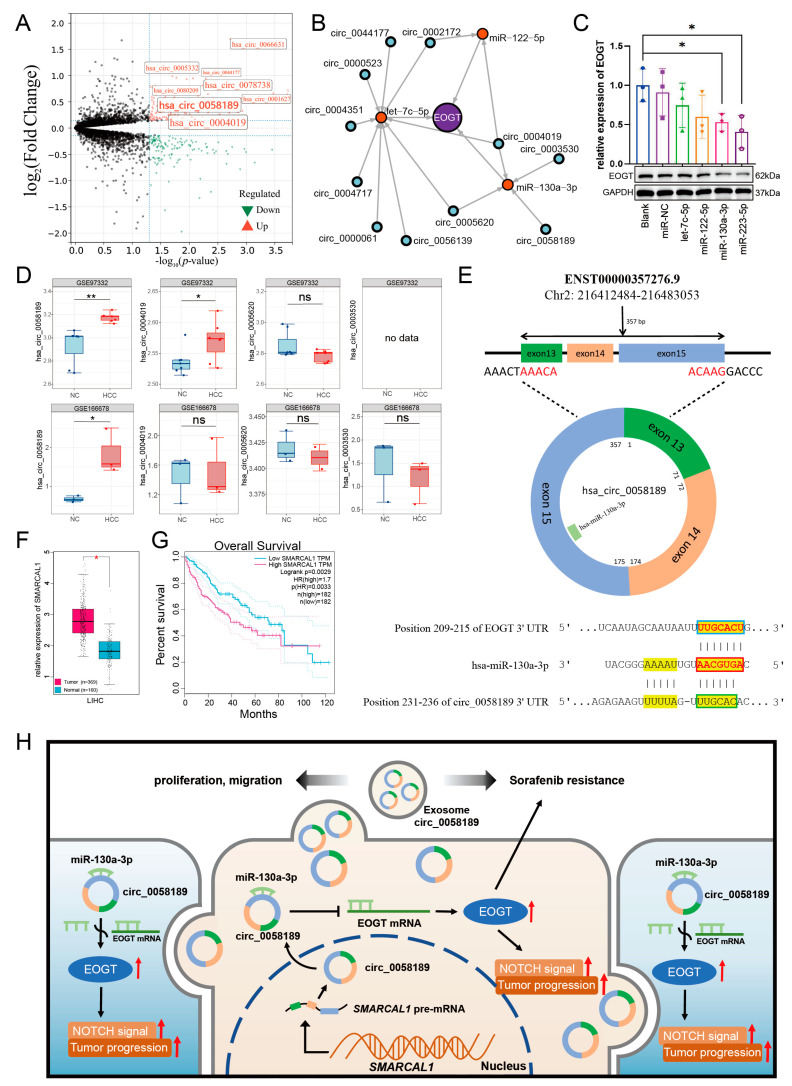
Potential critical role of circ_0058189/miR-130a/*EOGT* axis in HCC progression and sorafenib resistance. (**A**) Analysis of exosomal circRNAs from sorafenib-resistant HepG2 cells using dataset GSE101850, identifying 131 upregulated circRNAs. (**B**) Construction of a preliminary circRNA/miRNA/*EOGT* network using circMINE’s circRNA-miRNA prediction function to map interactions between the 131 circRNAs and the three key miRNAs (hsa-let-7c-5p, hsa-miR-122-5p, hsa-miR-130a-3p). (**C**) Western blot analysis showing that among the three key miRNAs, hsa-miR-130a-3p demonstrated the most significant regulatory effect on EOGT expression, establishing it as the central miRNA in the network. (**D**) External validation using GEO datasets GSE166678 and GSE97332, identifying hsa_circ_0058189 as consistently upregulated in both HCC tissues and plasma samples. (**E**) Bioinformatic analysis confirming molecular interactions within the axis: Targetscan identified a 7mer-m8 binding pattern between hsa-miR-130a-3p and *EOGT*, while circBase analysis revealed a non-canonical binding mode between hsa_circ_0058189 and hsa-miR-130a-3p. Red box represents the seed region, blue box represents the complementary region of *EOGT*, and green box represents the complementary region of circ_0058189. (**F**,**G**) Analysis of circ_0058189 host gene *SMRACAL1* in the TCGA database reveals significant upregulation in HCC, correlating with poor overall survival. (**H**) A model where circ_0058189 functions as a molecular sponge for hsa-miR-130a-3p, leading to EOGT derepression and contributing to HCC progression and sorafenib resistance, red upward arrows indicate upregulated protein expression, pathway activation, or HCC progression; * *p* < 0.05, ** *p* < 0.01, ns, not significant.

We then analyzed exosomal circRNAs from sorafenib-resistant HepG2 cells using dataset GSE101850. Comparing three sorafenib-resistant versus three sensitive samples (*p* < 0.05), we identified 131 upregulated circRNAs (Figure 9A, Table S7). Using circMINE’s circRNA-miRNA prediction function, we mapped interactions between the 131 circRNAs and our three key miRNAs (Table 2). This analysis enabled the construction of a preliminary circRNA/miRNA/*EOGT* network (Figure 9B).

### 3.12. Potential Critical Role of circ_0058189/miR-130a/EOGT Axis in HCC Progression and Sorafenib Resistance

To validate the functional relevance of these interactions, we performed Western blot analysis to assess the interference efficiency of the three key miRNAs on *EOGT* expression. Among the three key miRNAs, hsa-miR-130a-3p demonstrated the most significant regulatory effect, establishing it as the central miRNA in our network (Figure 9C).

Further refinement of the network focused on circRNAs interacting with hsa-miR-130a-3p. External validation using GEO datasets GSE166678 (plasma samples from three HCC patients vs. three healthy controls) and GSE97332 (seven HCC tissues vs. seven adjacent normal tissues) identified hsa_circ_0058189 as consistently upregulated in both HCC tissues and plasma samples (Figure 9D). Analysis of its host gene *SMRACAL1* in the TCGA database revealed significant upregulation in HCC, correlating with poor overall survival (Figure 9F,G) and suggesting *SMRACAL1* overexpression as a potential driver of hsa_circ_0058189 upregulation in HCC.

Bioinformatic analysis confirmed molecular interactions within the axis: Targetscan identified a 7mer-m8 binding pattern between hsa-miR-130a-3p and *EOGT*, while circBase analysis revealed a non-canonical binding mode between hsa_circ_0058189 and hsa-miR-130a-3p (Figure 9E). These findings support a model where hsa_circ_0058189 functions as a molecular sponge for hsa-miR-130a-3p, leading to *EOGT* derepression and contributing to HCC progression and sorafenib resistance (Figure 9H).

This regulatory axis is particularly prominent in HCC samples, suggesting its potential critical role in both HCC progression and therapeutic resistance. The identification of this axis provides a theoretical foundation for understanding HCC pathogenesis and developing novel therapeutic targets.

## 4. Discussion

EOGT, an endoplasmic reticulum-localized glycosyltransferase, catalyzes the addition of *O*-GlcNAc to proteins with EGF-like domains, thereby modulating their functional properties. Among the known *O*-GlcNAc glycosyltransferases, OGT and EOGT are the most well characterized. While OGT exhibits a broad substrate range, EOGT is specialized in modifying membrane-associated proteins. Previous research has established that OGT regulates iron metabolism and ferroptosis via dynamic intracellular *O*-GlcNAcylation [47]. In contrast, the role of EOGT-mediated *O*-GlcNAcylation in disease pathogenesis remains poorly understood. EOGT deficiency has been linked to genetic disorders, including Adams–Oliver syndrome, primarily through its modification of NOTCH receptors and subsequent regulation of downstream signaling pathways [48]. Furthermore, EOGT has been implicated in HCC, pancreatic cancer, and autoimmune liver diseases [11,12,49]; however, the underlying molecular mechanisms remain elusive. Notably, EOGT has recently been identified as a protein marker of migrasomes [50], a novel type of extracellular vesicle-like organelle generated during cell migration and a hallmark of malignant tumor behavior. Based on these findings, our study aimed to systematically analyze the expression, prognostic relevance, and functional roles of *EOGT* across various cancer types. Through a series of experimental validations, we identified EOGT as a novel driver of tumorigenesis in HCC. *EOGT* is significantly upregulated in HCC tissues and strongly correlates with poor patient prognosis. Furthermore, EOGT enhances the proliferation and migration capabilities of the HCC cell line. Notably, our study identifies EOGT as a key regulator in HCC progression and sorafenib resistance. Through a ceRNA network centered on *EOGT*, we uncovered the hsa_circ_0058189/miR-130a/*EOGT* axis as a critical regulatory mechanism. hsa_circ_0058189 functions as a molecular sponge for miR-130a-3p, effectively sequestering this miRNA and alleviating its inhibitory effect on *EOGT* expression. This axis is associated with poor prognosis and therapeutic resistance, providing insights into HCC pathogenesis and potential therapeutic targets.

In pan-cancer analysis, our findings align with recent studies demonstrating the upregulation of *EOGT* in multiple cancer types, including HCC and PAAD [11,12]. However, unlike the expected result, *EOGT* mRNA levels were downregulated in BRCA and COAD tumor tissues compared to their normal counterparts, while no significant differences were observed at the protein level. This discrepancy may be attributed to post-transcriptional and post-translational modifications of EOGT. For instance, in lung adenocarcinoma cells, METTL3, through its m^6^A methyltransferase activity, mediates the m^6^A modification of JUNB mRNA, which enhances mRNA stability and promotes its nuclear translocation as a transcription factor to activate downstream gene expression. m^6^A modification can compensate for low transcriptional levels by increasing mRNA stability and translation efficiency [51]. In our study, *EOGT* was found to be associated with the expression of multiple RNA modification-related genes, suggesting that the observed mRNA–protein discrepancy may result from post-transcriptional modifications. Additionally, in HCC, the E3 ubiquitin ligase TRIM21 targets β-catenin for polyubiquitination, reducing its levels and suppressing Wnt signaling, thereby inhibiting tumor cell proliferation and migration. However, TRIM21 downregulation in some HCC samples leads to insufficient β-catenin ubiquitination, allowing its accumulation despite reduced mRNA levels due to an impaired degradation mechanism [52]. This results in aberrant activation of the Wnt/β-catenin signaling pathway, promoting tumor growth and progression. These findings suggest that post-translational modifications also contribute to the discrepancy between protein and mRNA expression levels. However, whether EOGT is regulated by post-translational modifications requires extensive experimental validation, providing a direction for future research into the mechanisms of EOGT in cancer.

Furthermore, the high diagnostic accuracy of *EOGT* in LIHC (AUC = 0.844) is consistent with recent studies highlighting its role as a potential biomarker in HCC [12]. Our survival analysis revealed that *EOGT* appears to function as a protective factor in SKCM prognosis while acting as a risk factor in GBMLGG, LGG, KIPAN, ACC, LAML, and LIHC. This dual role of *EOGT* may stem from the activation of distinct molecular pathways in different cancer types, highlighting the inherent complexity and heterogeneity of cancer biology. *EOGT* has also been identified as a potential pan-cancer biomarker for tumor angiogenesis, and several candidate drugs targeting EOGT have been identified through screening studies. Notably, among these candidates, drugs such as tretinoin, mercaptopurine, imatinib, and butein have demonstrated anti-tumor effects, targeting diverse pathways, including lipid metabolism, purine metabolism, and inflammatory mediators. This suggests that EOGT may promote cancer through its glycosylation activity by modulating multiple mechanisms.

ICIs, primarily targeting CTLA-4, PD-1, and PD-L1, represent the most widely used and effective form of immunotherapy in clinical practice. TMB and MSI are predictive biomarkers for ICI sensitivity, with tumors exhibiting high TMB or MSI being more likely to respond to ICIs [53]. In most cancer types analyzed in this study, *EOGT* expression positively correlated with immune checkpoint gene expression, but only a subset of cancers showed significant associations with TMB and MSI. Following ICI treatment, non-responders showed no significant changes in *EOGT* levels compared to baseline, while responders in some datasets exhibited lower *EOGT* levels, suggesting that high *EOGT* levels may suppress the efficacy of immunotherapy in certain cancer types.

Considering the regulatory role of EOGT in the immune microenvironment, we further investigated its association with key tumor signaling pathways, and the enrichment analysis of proteomics data revealed that high EOGT expression is associated with active EMT pathways and DNA damage response pathways. Transcriptomic enrichment analysis indicated that high EOGT expression is linked to the activation of pro-tumorigenic pathways, including EMT, angiogenesis, inflammatory responses, TNFα signaling via NFκB, and TGF-β signaling, as well as the suppression of oxidative phosphorylation. When evaluating six different therapy-related tumor subtypes, high *EOGT* expression was predominantly observed in the C6 subtype, characterized by TGF-β dominance. TGF-β, a multifunctional cytokine, plays a critical role in regulating cell proliferation, differentiation, apoptosis, and immune modulation [54]. TGF-β is a key driver of EMT, whose activation induces tumor cell morphological changes, reduced cell adhesion, and enhanced motility, promoting local invasion and distant metastasis. These findings align with our transcriptomic, proteomic, and single-cell sequencing enrichment analyses [55]. TGF-β also contributes to angiogenesis by stimulating the expression of vascular endothelial growth factor (VEGF) and other pro-angiogenic factors, thereby promoting neovascularization. In advanced tumors, a rich vascular network not only supplies essential nutrients for rapid growth but also facilitates cancer cell entry into the bloodstream, increasing the risk of distant metastasis [56]. Additionally, TGF-β aids tumor immune evasion by modulating immunosuppressive states, such as increasing regulatory T-cell (Treg) numbers or function and reducing effector T-cell activity [57]. Therefore, the mechanism by which EOGT modulates multiple pathways through TGF-β to influence tumorigenesis remains unexplored, providing a direction for future research.

Endothelial cells play a pivotal role in the tumor microenvironment, not only supporting tumor progression through angiogenesis—providing essential nutrients and oxygen while removing metabolic waste—but also facilitating metastasis by enabling tumor cell proliferation within blood vessels. Additionally, endothelial cells contribute to immune regulation; for example, in HCC, tumor endothelial cells (TECs) can induce CD8^+^ T-cell exhaustion through GPNMB expression, thereby impairing the immune system’s ability to combat tumors [58]. In this study, both transcriptomic and single-cell transcriptomic data revealed a positive correlation between high *EOGT* expression and endothelial cell abundance. Additionally, transcriptomic, single-cell, and spatial transcriptomic data consistently indicated elevated *EOGT* expression in tumor cells. Knockdown and overexpression experiments in HCC cells further confirmed the critical role of EOGT in promoting malignant phenotypes. Therefore, the integration of potential EOGT inhibitors with current therapeutic regimens has the potential to augment treatment strategies by elevating tumor cell drug sensitivity, particularly in the context of current resistance to TKI-based medications.

The treatment landscape for HCC has evolved significantly, with novel targeted agents (e.g., lenvatinib) and ICIs (e.g., atezolizumab + bevacizumab) now serving as first-line therapies. Nevertheless, sorafenib—the first FDA-approved targeted drug for advanced HCC—remains an important therapeutic option. As a multi-kinase angiogenesis inhibitor, sorafenib functions through the dual inhibition of Raf kinase signaling and receptor tyrosine kinase (RTK) autophosphorylation (VEGFR, PDGFR, c-Kit, RET), thereby suppressing tumor progression and vascular development. However, the emergence of sorafenib resistance poses a major clinical challenge [59]. While the direct role of EOGT in sorafenib resistance remains uncharacterized, substantial evidence establishes its involvement in NOTCH pathway-mediated angiogenesis regulation. The angiogenic network comprises a dynamic balance of pro- and anti-angiogenic mediators, including growth factors, adhesion molecules, proteolytic enzymes, extracellular matrix components, transcriptional regulators, angiopoietins, angiostatin, endostatin, and interleukins. These elements collectively activate transmembrane receptors to drive endothelial cell proliferation, survival, and neovascularization. Of particular relevance, NOTCH receptors—subject to EOGT-mediated modification—represent one of the critical components of this signaling architecture.

Our mechanistic hypothesis derives from two critical considerations: (1) NOTCH receptors constitute a distinct non-RTK protein class unaffected by sorafenib’s primary targets, and (2) the compensatory activation of alternative pro-angiogenic pathways—recognized as the principal driver of sorafenib resistance—may include NOTCH pathway upregulation following the suppression of dominant angiogenic signaling. This biological context positions EOGT as a potential key mediator of resistance-associated compensatory angiogenesis. Consequently, the regulatory axis involving *EOGT* may indeed be pivotal in the development of sorafenib resistance.

CircRNAs, particularly those enriched in exosomes, show promise as cancer biomarkers and therapeutic targets [60]. Emerging evidence suggests that exosomal circRNAs from drug-resistant cells can mediate therapy resistance [61]. For instance, circ_002136 promotes HCC progression through the miR-19a-3p/*RAB1A* axis [62], while tumor-derived exosomal circUHRF1 induces NK cell exhaustion and PD-1 resistance [63]. In this context, our findings demonstrate significant upregulation of circ_0058189 in HCC tissues, plasma, and exosomes from sorafenib-resistant HepG2 cells, suggesting its potential role in therapeutic resistance.

The tumor-suppressive role of miR-130a-3p has been established across multiple cancers, including HCC [64,65,66,67,68]. Its downregulation in HCC tissues correlates with poor prognosis, consistent with Wang’s findings linking miR-130a-3p deficiency to COX7RP-mediated ROS/NF-κB activation and Hu’s work demonstrating its regulation of HIF1A-driven glycolysis [66,69]. Our bioinformatic and experimental analyses reveal that circ_0058189 acts as a molecular sponge for miR-130a-3p, with predicted binding sites supporting this interaction.

Notably, EOGT emerges as a critical oncogenic target in HCC. Our multi-modal validation confirms *EOGT* as a direct target of miR-130a-3p, establishing the circ_0058189/miR-130a-3p/*EOGT* regulatory axis. The consistent upregulation of circ_0058189 in HCC tissues, plasma, and exosomes from sorafenib-resistant HepG2 cells highlights its potential as a dual diagnostic and therapeutic resistance biomarker.

While our study highlights the critical role of exosome-mediated circ_0058189 in regulating the miR-130a-3p/*EOGT* axis during HCC progression and sorafenib resistance, several limitations should be acknowledged. First, although TCGA and other public datasets provide valuable insights, and our core findings in HCC are supported by substantial sample sizes (TCGA-LIHC: 369 tumor vs. 50 normal samples, as shown in Table S1), the limited sample size may restrict the generalizability of our findings. Future studies should validate these results in larger, independent cohorts. Second, while we employed multi-layered bioinformatic analyses and preliminary experiments to explore the circ_0058189/miR-130a-3p/*EOGT* axis, more comprehensive functional validation in cellular and animal models is needed to elucidate its mechanistic and clinical significance. For instance, we could employ RNA pull-down combined with dual-luciferase reporter assays to validate the specific binding between circ_0058189 and miR-130a-3p; utilize AGO2-RIP experiments to confirm the direct targeting of *EOGT* mRNA by miR-130a-3p; and establish patient-derived xenograft (PDX) models coupled with liquid biopsy cohorts to verify the clinical translational potential of this axis in sorafenib resistance in HCC. Third, HCC pathogenesis involves complex interactions among multiple molecular pathways. Focusing on a single axis may not fully capture the disease’s complexity. Integrating multi-omics data to construct a more comprehensive molecular network is essential for a deeper understanding of HCC mechanisms. Fourth, our findings do not exclude EOGT’s pleiotropic functions in therapy resistance. Future studies must prioritize the systematic characterization of EOGT-*O*-GlcNAc substrates in diverse treatment contexts using approaches such as CRISPR-based screens coupled with drug sensitivity profiling. Fifth, while our study provides insights into *EOGT* as a pan-cancer angiogenesis marker, it is important to note that *EOGT* is not universally upregulated across all tumor types. Future investigations integrating single-cell omics with spatial proteomics could fully resolve the spatiotemporal heterogeneity of EOGT expression within tumor-associated endothelial niches, potentially addressing this limitation. Finally, our study primarily focused on exosomes derived from HepG2 cells. Given the heterogeneity of the tumor microenvironment, exosomes from other cell types may exhibit distinct functional properties. Future investigations should explore the roles of exosomes from diverse cellular sources in HCC progression and therapy resistance.

## 5. Conclusions

In summary, through pan-cancer analyses, we identified *EOGT* as a key oncogene significantly upregulated in HCC and associated with poor prognosis. Functional experiments demonstrated that EOGT drives tumor progression by enhancing proliferation and migration in HCC. Mechanistically, we revealed a novel circ_0058189/miR-130a-3p/*EOGT* axis, illustrating how the ceRNA network regulates *EOGT* to promote carcinogenesis and drug resistance. Furthermore, EOGT’s roles in immune modulation, angiogenesis, and DNA repair underscore its multifaceted contributions to cancer biology. Notably, EOGT emerged as a potential pan-cancer biomarker and a mediator of chemotherapeutic resistance, highlighting its therapeutic relevance. These findings deepen our understanding of EOGT’s oncogenic functions and provide a foundation for developing targeted therapies against HCC and other cancers. Future studies should focus on EOGT’s post-translational regulation and clinical translation.

## Figures and Tables

**Table 1:** The data from the dbDEMC database were utilized to further validate and obtain five miRNAs with more robust downregulated expression.

**miRNA ID**	**Source ID**	**Design**	**logFC**	**Experiment ID**
hsa-let-7c-5p	GSE115016	cancer vs. normal	−0.8	EXP00501
	SRP049590	cancer vs. normal	−0.69	EXP00719
hsa-miR-122-5p	GSE147889	cancer vs. normal	−0.69	EXP00576
hsa-miR-130a-3p	GSE147889	cancer vs. normal	−1.23	EXP00576
	E_MTAB_4170	cancer vs. normal	−1.3	EXP00627
	SRP049590	cancer vs. normal	−0.98	EXP00719
hsa-miR-223-5p	SRP049590	cancer vs. normal	−1.1	EXP00719
hsa-miR-542-5p	GSE21362	cancer vs. normal	−1.2	EXP00117
	GSE40744	cancer vs. normal	−0.81	EXP00213
	GSE36915	cancer vs. normal	−0.68	EXP00221
	GSE147889	cancer vs. normal	−0.81	EXP00576

**Table 2:** Potential circRNAs screened from the circMINE database.

**miRNA**	**circRNA**
hsa-let-7c-5p	hsa_circ_0044177
	hsa_circ_0005620
	hsa_circ_0002172
	hsa_circ_0000523
	hsa_circ_0004351
	hsa_circ_0004717
	hsa_circ_0000061
	hsa_circ_0056139
	hsa_circ_0004019
hsa-miR-122-5p	hsa_circ_0002172
	hsa_circ_0004019
hsa-miR-130a-3p	hsa_circ_0005620
	hsa_circ_0058189
	hsa_circ_0004019
	hsa_circ_0003530

## Data Availability

All data are provided in the Supplementary Materials or are available from the corresponding author upon reasonable request.

## Supplementary Materials

The following supporting information can be downloaded at: https://www.mdpi.com/article/10.3390/biomedicines13040773/s1, Figure S1. A Relative EOGT expression levels across various cell lines, obtained from the Cancer Cell Line Encyclopedia (CCLE). B EOGT expression evaluated across 33 tumor types based on gender, clinical stage, grade, and TNM classification; * *p* < 0.05, ** *p* < 0.01, *** *p* < 0.001, **** *p* < 0.0001. Full terminology for abbreviations is provided in Table S1. Figure S2. Receiver Operating Characteristic Curve (ROC) curves demonstrating EOGT’s potential as a diagnostic biomarker for various cancers. Full terminology for abbreviations is provided in Table S1. Figure S3. Kaplan Meier curves validating the associations between EOGT expression and survival outcomes (OS, DSS, DFI, PFI) in different cancers, indicating that high EOGT expression generally correlates with poor prognosis in LGG, KIPAN, ACC, LAML, and LIHC. Full terminology for abbreviations is provided in Table S1. Figure S4. Inverse correlation between EOGT expression and promoter methylation in 30 cancer types. Full terminology for abbreviations is provided in Table S1. Figure S5. Reduced methylation of EOGT predicting shorter survival in LGG and THYM. Full terminology for abbreviations is provided in Table S1. Figure S6. Gene Set Enrichment Analysis (GSEA) linking high EOGT expression to the activation of pro-cancer pathways, including EMT, angiogenesis, inflammatory response, and TNFα signaling via NF-κB while suppressing oxidative phosphorylation. Full terminology for abbreviations is provided in Table S1. Figure S7. A Scatter plots illustrating the strongest correlations between ESTIMATE scores and EOGT expression levels in the top 6 cancer types. B EOGT upregulation in the C6 immunological subtype of BRCA, COAD, HNSC, KIRC, LUAD, LUSC, STAD, and THCA, suggesting a potential functional relationship with TGF-β signaling activity. Full terminology for abbreviations is provided in Table S1. Figure S8. Pan-cancer analysis of correlations between EOGT and immune-related genes; * *p* < 0.05. Full terminology for abbreviations is provided in Table S1. Figure S9. A Comparison of EOGT expression in samples before and after treatment with immune checkpoint inhibitors (ICIs) in vivo using the TISMO database. B Comparison of EOGT expression in tumor cell lines treated with cytokines in vitro using the TISMO database; * *p* < 0.05, ** *p* < 0.01, *** *p* < 0.001. Figure S10. Assessment of the correlation between EOGT expression and infiltration of various immune cell types to understand the association between EOGT expression and cancer-related immune activity. Full terminology for abbreviations is provided in Table S1; * *p* < 0.05, ** *p* < 0.01, *** *p* < 0.001, **** *p* < 0.0001. Figure S11. A-H Spatial transcriptomics revealing overlapping EOGT expression patterns with tumor cells in SKCM and GBM. I, J CancerSEA analysis linking EOGT to angiogenesis, cell differentiation, EMT, inflammation, metastasis, and stemness while showing weak or negative correlations with cell cycle, DNA repair, and DNA damage. Full terminology for abbreviations is provided in Table S1; * *p* < 0.05, ** *p* < 0.01, *** *p* < 0.001, **** *p* < 0.0001. Figure S12. Association of EOGT expression with drug resistance and identification of potential EOGT inhibitors. A Heatmap illustrating the association of high EOGT expression with resistance to multiple chemotherapeutic agents, particularly tyrosine kinase inhibitors, across the GDSC1/2, CTRP, and PRISM databases. Drug sensitivity was evaluated using IC50 and AUC. Representative examples include Afatinib, Lapatinib, Gefitinib, and Selumetinib, showing significant correlations in multiple datasets. B Heatmap summarizing the three most inhibitory drugs for each cancer type, as identified through CMAP analysis. Arachidonyltrifluoromethane and W.13 each appear 9 times, while MK-886 and X4.5.dianilinophthalimide each appear 8 times, suggesting their potential to target EOGT or its synergistic pathways across multiple cancer types. Full terminology for abbreviations is provided in Table S1. Table S1. TCGA cancer classification and sample inventory. Table S2. The miRNA mimics and their sequences. Table S3. All information of antibodies. Table S4. Fifteen EOGT clinical-relevant AS events on OncoSplicing. Table S5. Results of the top 20 miRNAs targeting EOGT screened from the mirDIP database. Table S6. Results of the 79 miRNAs targeting EOGT screened from the mirDIP and miRwalk databases. Table S7. The 131 upregulated circRNAs and name conversion results from the GSE101850 dataset.

## Abbreviations

SLC7A11: Solute carrier family 7 member 11; NRF2: Nuclear factor erythroid-2; ChIP: chromatin immunoprecipitation; OS: overall survival; PFS: progression-free survival; DFS: disease-free survival; DSS: disease-specific survival; OD: optical density; ROS: reactive oxygen species; HCC: hepatocellular carcinoma; CRC: colorectal cancer; GlcNAc: *O*-linked *N*-acetylglucosamine; *O*-GlcNAcylation: *O*-linked β-*N*-acetylglucosamine modification; YAP: Yes-associated protein; YY1: Yin-yang 1 transcription factor; SLC22A15: Solute carrier family 22 member 15; AANAT: Arylalkylamine *N*-acetyltransferase; *UDP*-GlcNAc: Uridine diphosphate *N*-acetylglucosamine; PD-L1: Programmed Death-Ligand 1; OGT: *O*-GlcNAc transferase; OGA: *O*-GlcNAcase; GSH: Glutathione; NOTCH: Notch receptor; HEY1: Hes related family bHLH transcription factor 1; HES1: Hes family bHLH transcription factor 1; PINK1: PTEN-induced putative kinase 1; CAMS: Chinese Academy of Medical Sciences; PBS: Phosphate-buffered solution; DMEM: Dulbecco’s modified Eagle’s medium; FBS: fetal bovine serum; DAPT: Notch receptor inhibitor; DMSO: dimethyl sulfoxide; shRNA: short hairpin RNA; CCK-8: Cell Counting Kit-8; SD: standard deviation; ICGC: International Cancer Genome Consortium; TCGA: The Cancer Genome Atlas; GEO: Gene Expression Omnibus; HPA: Human Protein Atlas; EGF: epidermal growth factor; LRP8: Low-Density Lipoprotein Receptor-Related Protein 8; HDACs: histone deacetylases; EMT: epithelial-to-mesenchymal transition.
