# Supplementary material for: Migrasome Marker Epidermal Growth Factor Domain-Specific O-GlcNAc Transferase: Pan-Cancer Angiogenesis Biomarker and the Potential Role of circ_0058189/miR-130a-3p/EOGT Axis in Hepatocellular Carcinoma Progression and Sorafenib Resistance
Source: Biomedicines. 2025 Mar 22;13(4):773. doi: 10.3390/biomedicines13040773 (PMC12024942; doi:10.3390/biomedicines13040773)
Supplement: Supplementary file 1 [file biomedicines-13-00773-s001.zip › biomedicines-3506755-supplementary.pdf]

Supplementary Materials

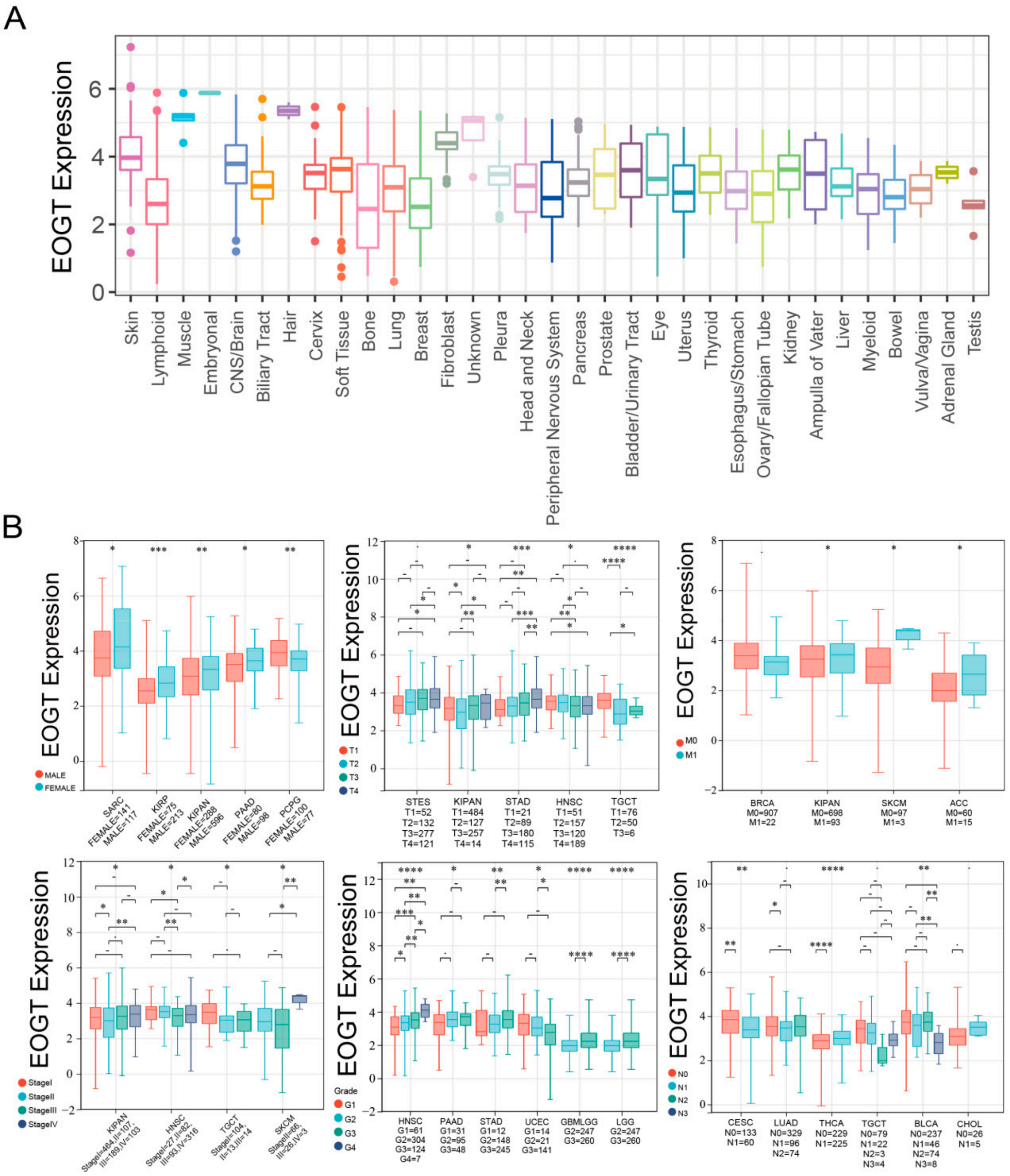

Figure S1. A Relative EOGT expression levels across various cell lines, obtained from the Cancer Cell Line Encyclopedia (CCLE). **B** EOGT expression evaluated across 33 tumor types based on gender, clinical stage, grade, and TNM classification; **p*<0.05, ***p*<0.01, ****p*<0.001, *****p*<0.0001. Full terminology for abbreviations is provided in Table S1.

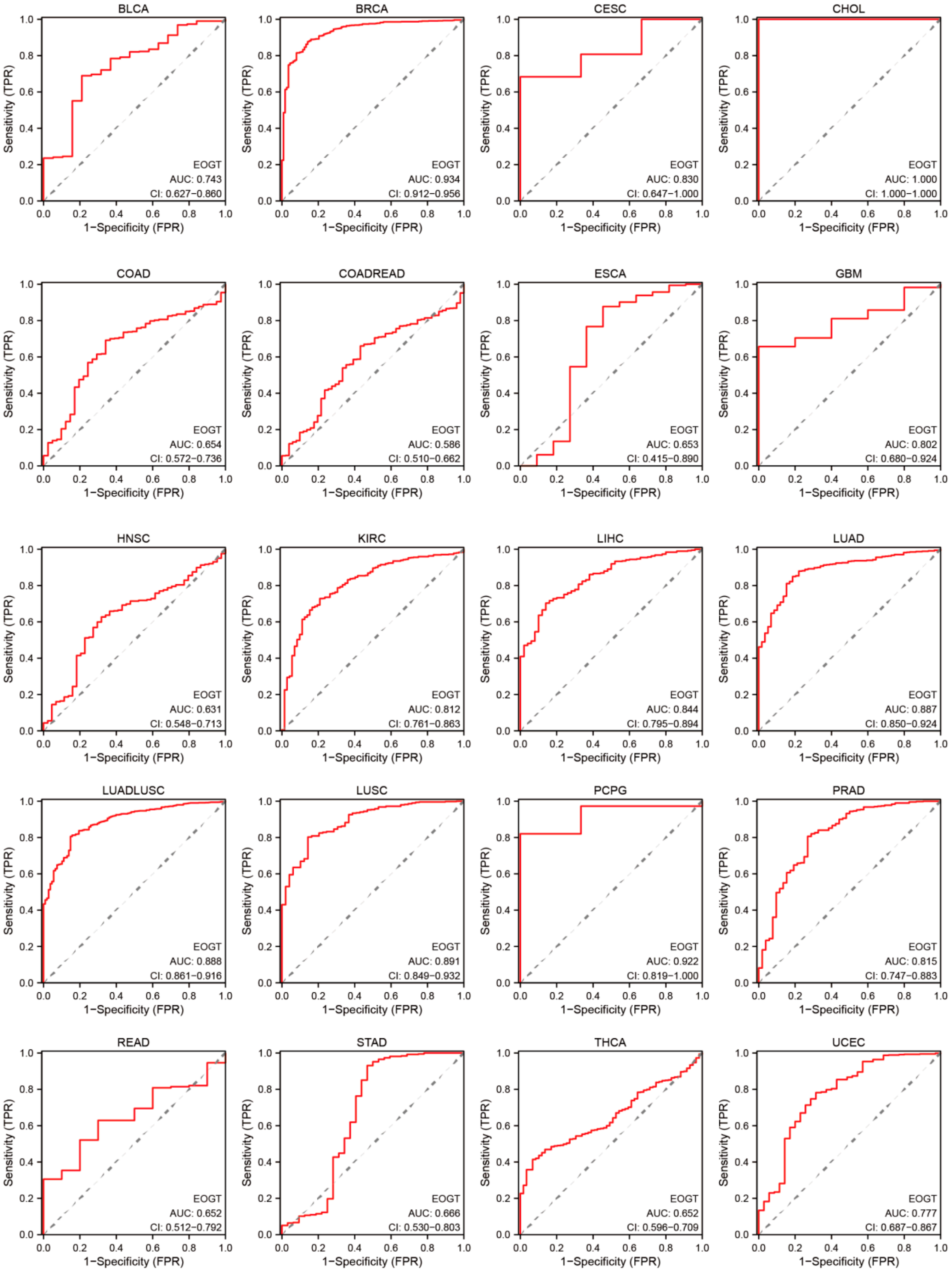

Figure S2. Receiver Operating Characteristic Curve (ROC) curves demonstrating EOGT's potential as a diagnostic biomarker for various cancers. Full terminology for abbreviations is provided in Table S1.

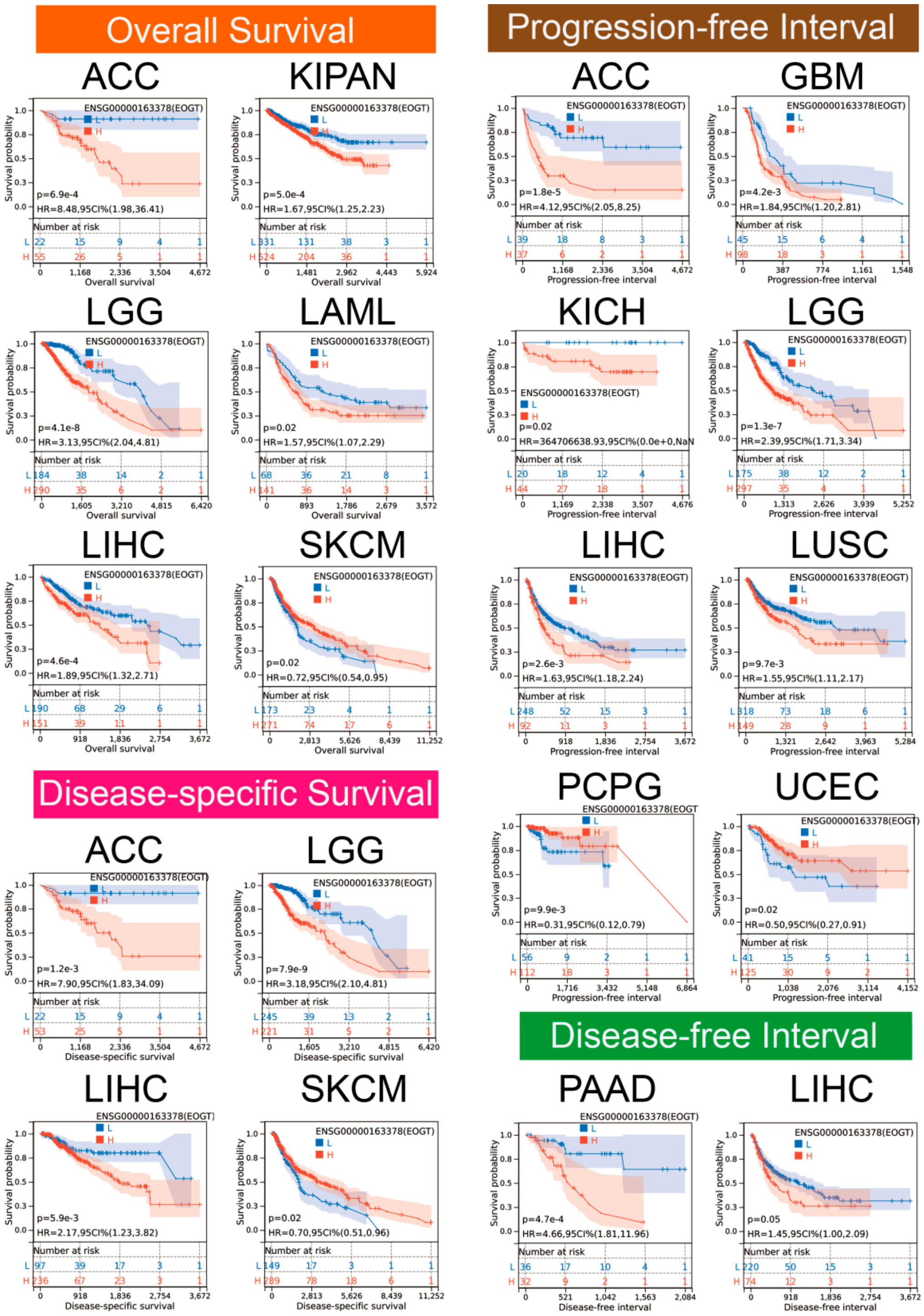

Figure S3. Kaplan Meier curves validating the associations between EOGT expression and survival outcomes (OS, DSS, DFI, PFI) in different cancers, indicating that high EOGT expression generally correlates with poor prognosis in LGG, KIPAN, ACC, LAML, and LIHC. Full terminology for abbreviations is provided in Table S1.

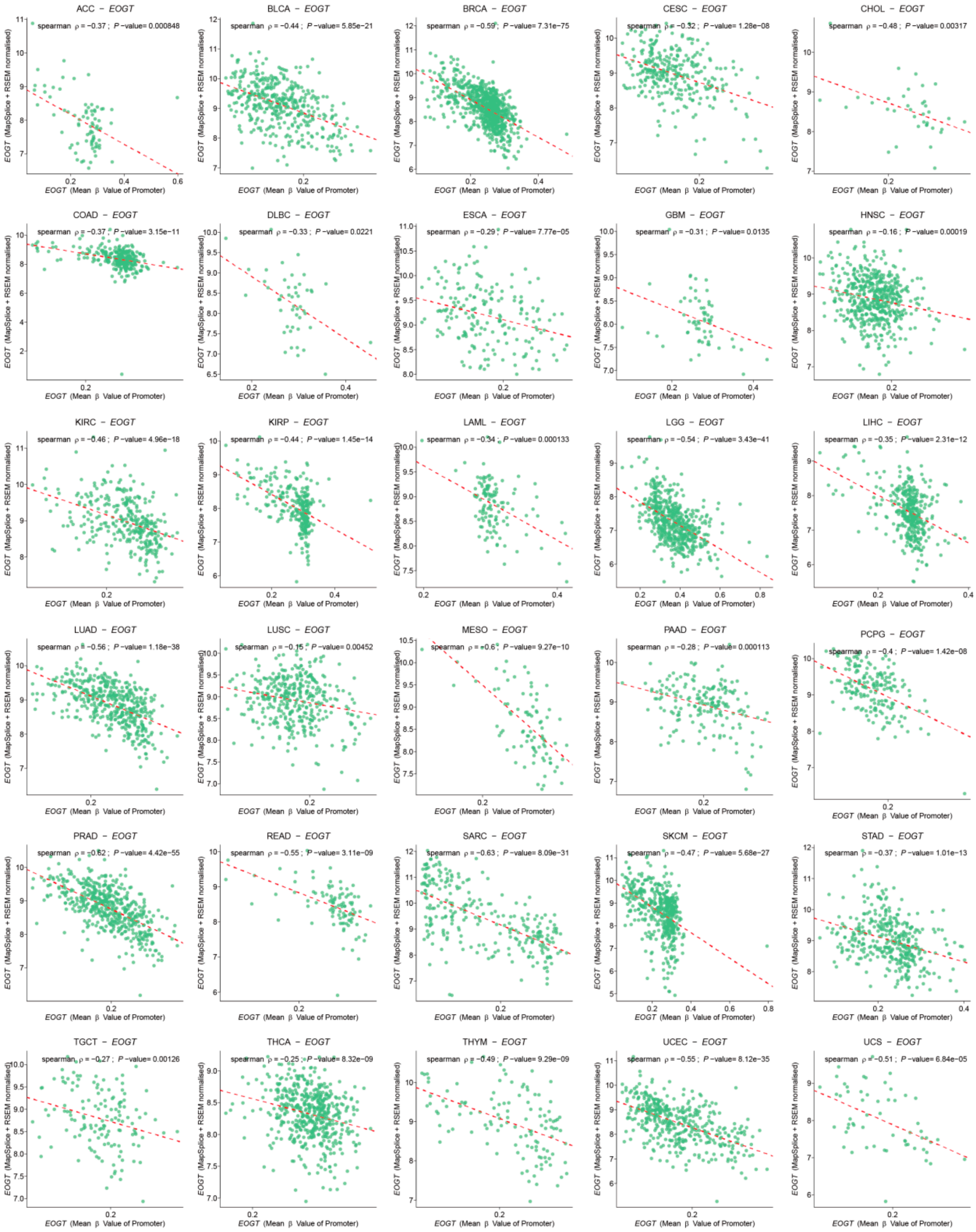

Figure S4. Inverse correlation between EOGT expression and promoter methylation in 30 cancer types. Full terminology for abbreviations is provided in Table S1.

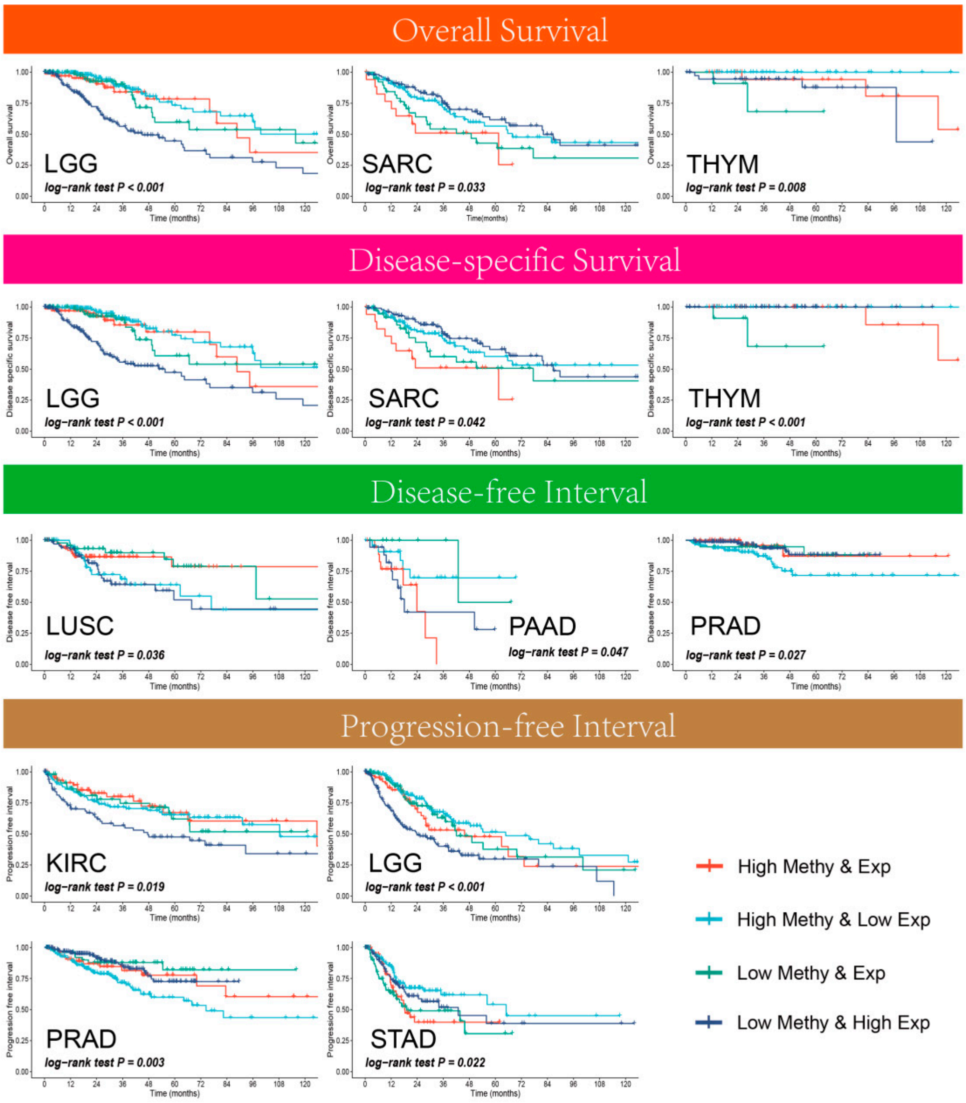

Figure S5. Reduced methylation of EOGT predicting shorter survival in LGG and THYM. Full terminology for abbreviations is provided in Table S1.

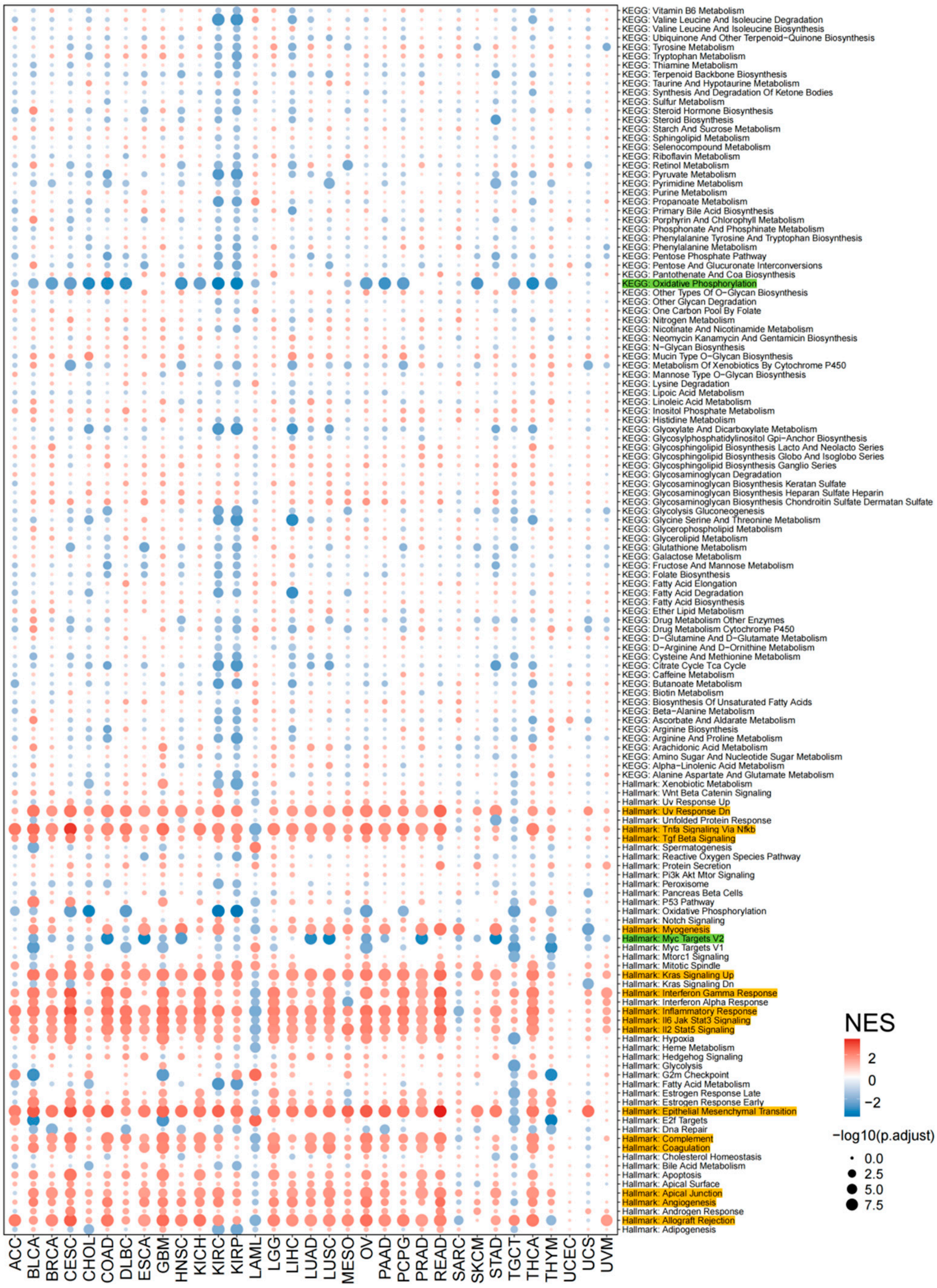

Figure S6. Gene Set Enrichment Analysis (GSEA) linking high EOGT expression to the activation of pro-cancer pathways, including EMT, angiogenesis, inflammatory response, and TNFα signaling via NF-κB while suppressing oxidative phosphorylation. Full terminology for abbreviations is provided in Table S1.

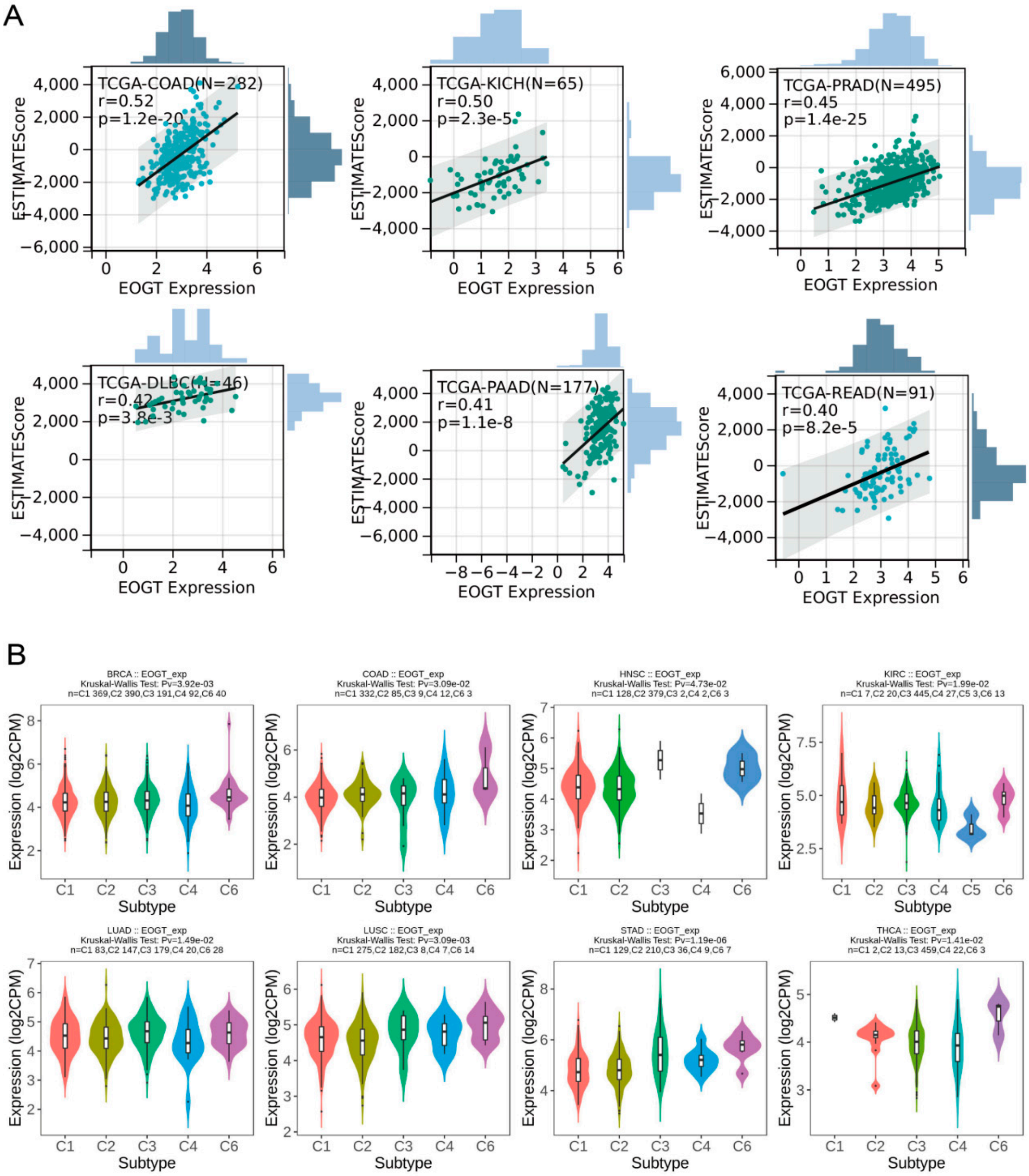

Figure S7. A Scatter plots illustrating the strongest correlations between ESTIMATE scores and EOGT expression levels in the top 6 cancer types. **B** EOGT upregulation in the C6 immunological subtype of BRCA, COAD, HNSC, KIRC, LUAD, LUSC, STAD, and THCA, suggesting a potential functional relationship with TGF-β signaling activity. Full terminology for abbreviations is provided in Table S1.

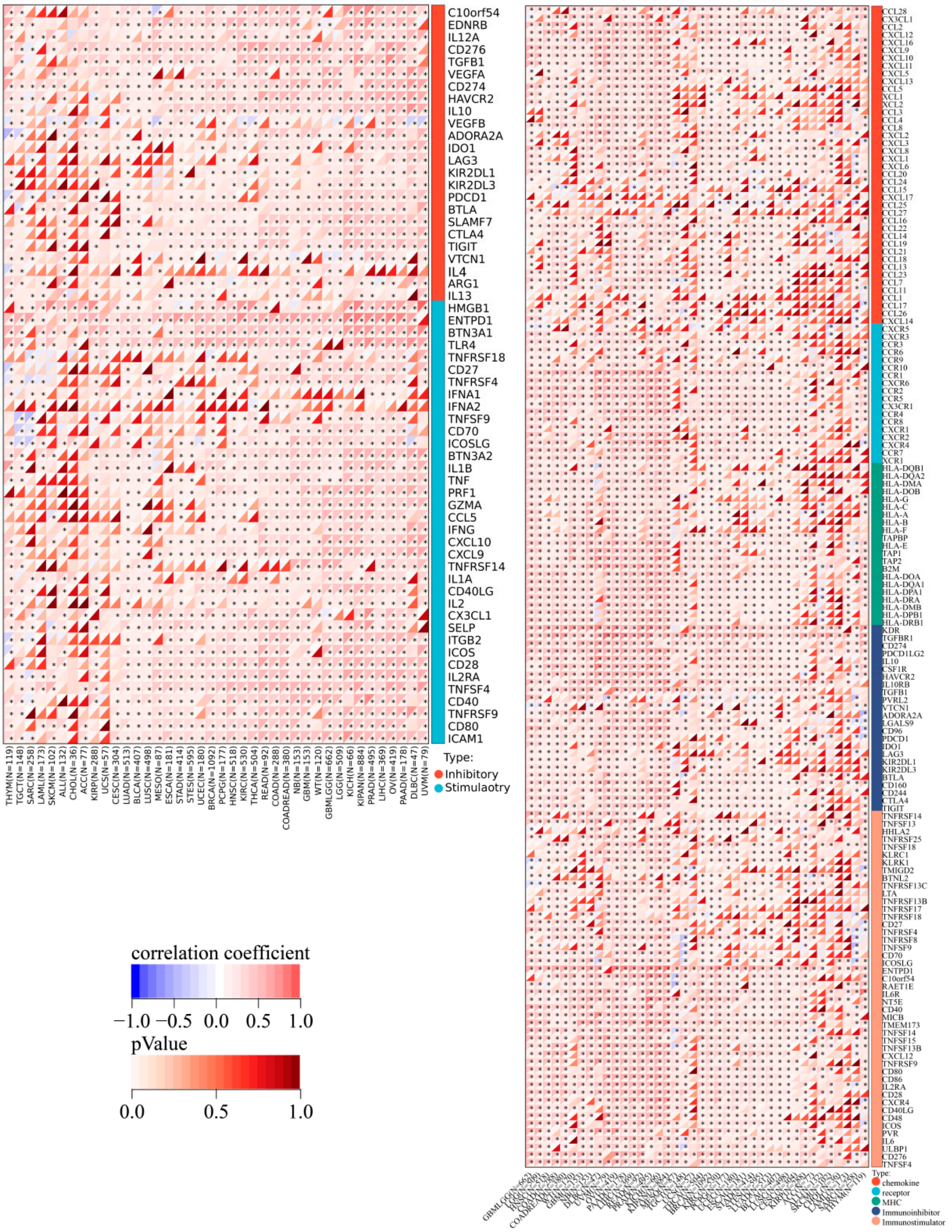

Figure S8. Pan-cancer analysis of correlations between EOGT and immune-related genes; **p*<0.05. Full terminology for abbreviations is provided in Table S1.

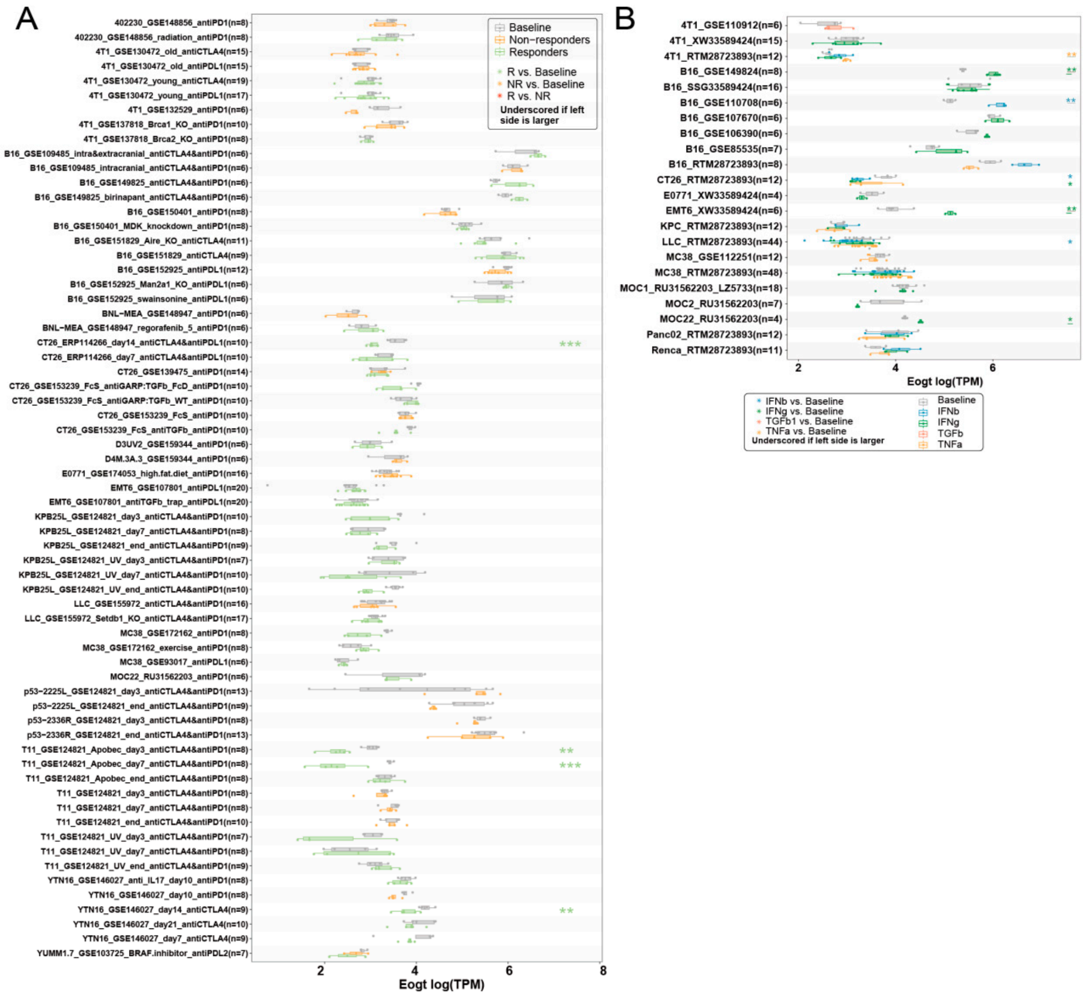

Figure S9. A Comparison of EOGT expression in samples before and after treatment with immune checkpoint inhibitors (ICIs) in vivo using the TISMO database. **B** Comparison of EOGT expression in tumor cell lines treated with cytokines in vitro using the TISMO database; **p*<0.05, ***p*<0.01, ****p*<0.001.

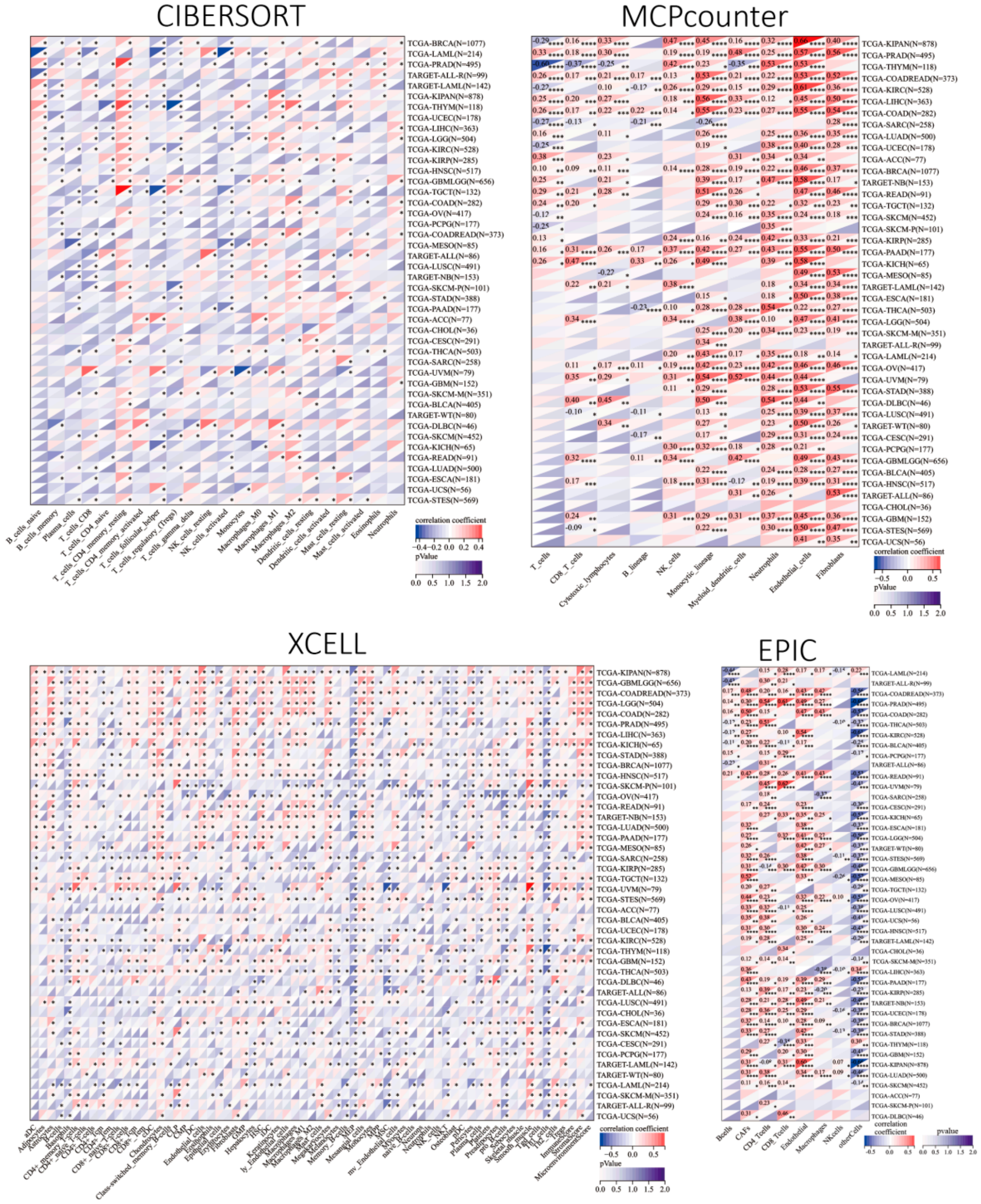

Figure S10. Assessment of the correlation between EOGT expression and infiltration of various immune cell types to understand the association between EOGT expression and cancer-related immune activity. Full terminology for abbreviations is provided in Table S1; **p*<0.05, ***p*<0.01, ****p*<0.001, *****p*<0.0001.

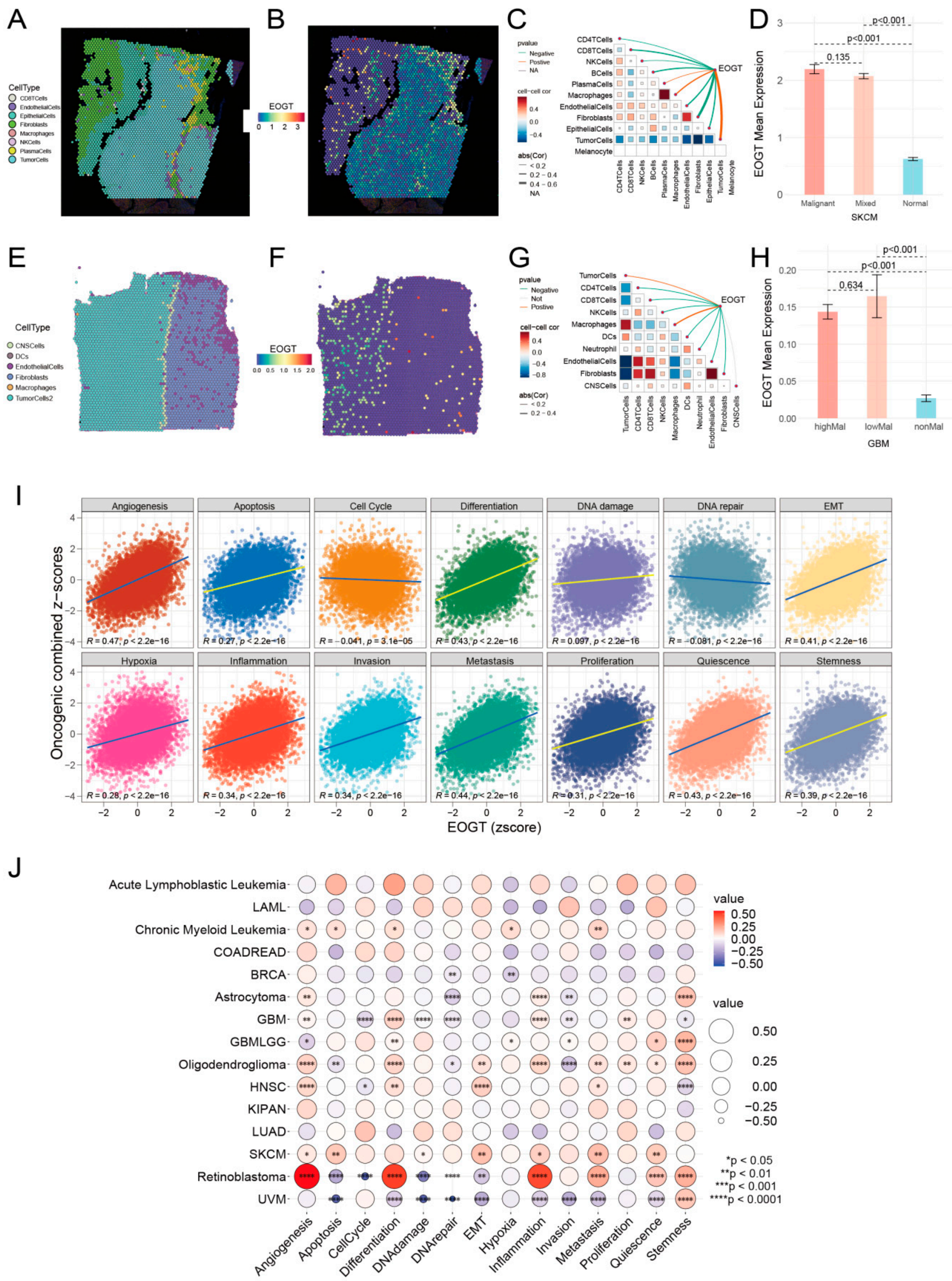

Figure S11. A-H Spatial transcriptomics revealing overlapping EOGT expression patterns with tumor cells in SKCM and GBM. **I, J** CancerSEA analysis linking EOGT to angiogenesis, cell differentiation, EMT, inflammation, metastasis, and stemness while showing weak or negative correlations with cell cycle, DNA repair, and DNA damage. Full terminology for abbreviations is provided in Table S1; **p*<0.05, ***p*<0.01, ****p*<0.001, *****p*<0.0001.

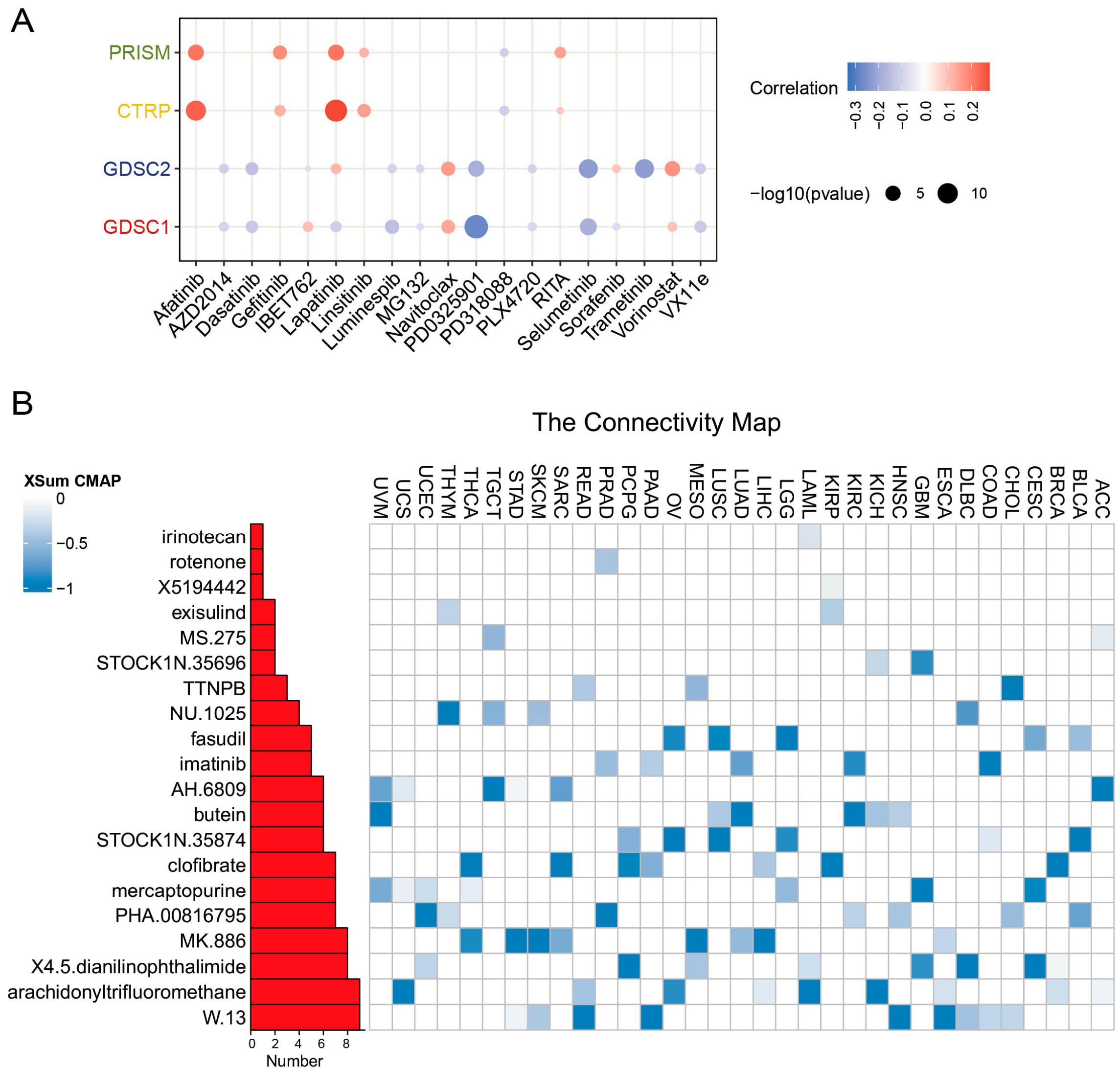

Figure S12. Association of EOGT expression with drug resistance and identification of potential EOGT inhibitors. **A** Heatmap illustrating the association of high EOGT expression with resistance to multiple chemotherapeutic agents, particularly tyrosine kinase inhibitors, across the GDSC1/2, CTRP, and PRISM databases. Drug sensitivity was evaluated using IC50 and AUC. Representative examples include Afatinib, Lapatinib, Gefitinib, and Selumetinib, showing significant correlations in multiple datasets. **B** Heatmap summarizing the three most inhibitory drugs for each cancer type, as identified through CMAP analysis. Arachidonyltrifluoromethane and W.13 each appear 9 times, while MK-886 and X4.5.dianilinophthalimide each appear 8 times, suggesting their potential to target EOGT or its synergistic pathways across multiple cancer types. Full terminology for abbreviations is provided in Table S1.

Table S1. TCGA cancer classification and sample inventory.

Abbreviation	Full name	Tumor Samples	Normal Samples
ACC	Adrenocortical Carcinoma	T=77	N=0
BLCA	Bladder Urothelial Carcinoma	T=407	N=19
BRCA	Breast Invasive Carcinoma	T=1092	N=113

CESC	Cervical Squamous Cell Carcinoma and Endocervical Adenocarcinoma	T=304	N=3
CHOL	Cholangiocarcinoma	T=36	N=9
COAD	Colon Adenocarcinoma	T=288	N=41
COADREAD	Colorectal Adenocarcinoma	T=380	N=51
DLBC	Lymphoid Neoplasm Diffuse Large B-cell Lymphoma	T=48	N=0
ESCA	Esophageal Carcinoma	T=181	N=13
GBM	Glioblastoma Multiforme	T=153	N=5
GBMLGG	Glioma (GBM + LGG)	T=662	N=5
HNSC	Head and Neck Squamous Cell Carcinoma	T=518	N=44
KICH	Kidney Chromophobe	T=66	N=129
KIPAN	Pan-Kidney Cohort (KICH + KIRC + KIRP)	T=884	N=129
KIRC	Kidney Renal Clear Cell Carcinoma	T=530	N=129
KIRP	Kidney Renal Papillary Cell Carcinoma	T=288	N=129
LAML	Acute Myeloid Leukemia	T=173	N=0
LGG	Brain Lower Grade Glioma	T=509	N=5
LIHC	Liver Hepatocellular Carcinoma	T=369	N=50
LUAD	Lung Adenocarcinoma	T=513	N=109
LUSC	Lung Squamous Cell Carcinoma	T=498	N=109
MESO	Mesothelioma	T=17	N=0
OV	Ovarian Serous Cystadenocarcinoma	T=419	N=0
PAAD	Pancreatic Adenocarcinoma	T=178	N=4
PCPG	Pheochromocytoma and Paraganglioma	T=177	N=3
PRAD	Prostate Adenocarcinoma	T=495	N=52
READ	Rectum Adenocarcinoma	T=92	N=10
SARC	Sarcoma	T=261	N=21
SKCM	Skin Cutaneous Melanoma	T=102	N=0
STAD	Stomach Adenocarcinoma	T=414	N=36
TGCT	Testicular Germ Cell Tumors	T=148	N=0
THCA	Thyroid Carcinoma	T=504	N=59
THYM	Thymoma	T=9	N=2
UCEC	Uterine Corpus Endometrial Carcinoma	T=180	N=23
UCS	Uterine Carcinosarcoma	T=57	N=0
UVM	Uveal Melanoma	T=129	N=0

Table S2. The miRNA mimics and their sequences.

miRNA ID	Accession number	sequences
hsa-let-7c-5p	MIMAT0000064	5′ -UGAGGUAGUAGGUUGUAUGGUU-3′
hsa-miR-122-5p	MIMAT0000421	5′ -UGGAGUGUGACAAUGGUGUUUG-3′
hsa-miR-130a-3p	MIMAT0000425	5′ -CAGUGCAAUGUUAAAAGGGCAU-3′
hsa-miR-223-5p	MIMAT0004570	5′ -CGUGUAUUUGACAAGCUGAGUU-3′
miR-NC	-	5′ -UUCUCCGAACGUGUCACGUTT-3′

Table S3. All information of antibodies.

Gene Symbol	Catalog Number	Source	Product Name
EOGT	ab190693	Abcam	anti-AER61 antibody [EPR12944]
HEY1	19929-1-AP	Proteintech	HEY1 Polyclonal antibody

SLC7A11	26864-1-AP	Proteintech	SLC7A11/xCT Polyclonal antibody
GAPDH	#2118	Cell Signaling	GAPDH(14C10)Rabbit mAb

Table S4. Fifteen EOGT clinical-relevant AS events on OncoSplicing.

Gene_Symbol	Splice_Type	Splice_Event	Project
EOGT	A5	alt_5prime_149865	SpIAdder
EOGT	A5	alt_5prime_149866	SpIAdder
EOGT	A5	alt_5prime_149869	SpIAdder
EOGT	ES	exon_skip_385667	SpIAdder
EOGT	ES	exon_skip_385670	SpIAdder
EOGT	IR	intron_retention_100969	SpIAdder
EOGT	AP	EOGT_AP_65559	SpliceSeq
EOGT	AP	EOGT_AP_65560	SpliceSeq
EOGT	ES	EOGT_ES_190946	SpliceSeq
EOGT	ES	EOGT_ES_190947	SpliceSeq
EOGT	ES	EOGT_ES_190948	SpliceSeq
EOGT	ES	EOGT_ES_191275	SpliceSeq
EOGT	ES	EOGT_ES_191276	SpliceSeq
EOGT	ES	EOGT_ES_65561	SpliceSeq
EOGT	ES	EOGT_ES_65562	SpliceSeq

ES, Exon Skipping; IR, Intron Retention; A5, Alternative 5′ Splice Site; A3, Alternative 3′ Splice Site; AP, Alternative Polyadenylation.

Table S5. Results of the top 20 miRNAs targeting EOGT screened from the mirDIP database.

Gene Symbol	Uniprot	microRNA	Integrated Score
EOGT	Q5NDL2	hsa-miR-148a-3p	0.890092391
		hsa-miR-152-3p	0.884545713
		hsa-miR-148b-3p	0.880114569
		hsa-miR-454-3p	0.873646079
		hsa-miR-301a-3p	0.872221875
		hsa-miR-98-5p	0.871887063
		hsa-miR-301b-3p	0.86408802
		hsa-let-7d-5p	0.851615544
		hsa-let-7g-5p	0.846192198
		hsa-let-7i-5p	0.843165545
		hsa-let-7c-5p	0.830131915
		hsa-let-7b-5p	0.828603018
		hsa-let-7e-5p	0.820467294
		hsa-miR-130a-3p	0.809915336
		hsa-miR-19a-3p	0.808790419
		hsa-let-7f-5p	0.807309619
		hsa-miR-130b-3p	0.801279403
		hsa-miR-19b-3p	0.799402704
		hsa-let-7a-5p	0.787809686
		hsa-miR-9-5p	0.732748216

Table S6. Results of the 79 miRNAs targeting EOGT screened from the mirDIP and miRwalk databases.

miRNA.ID	miRNA.ID	miRNA.ID	miRNA.ID
let-7a-5p	miR-149-3p	miR-320d	miR-557
let-7b-5p	miR-152-3p	miR-3688-3p	miR-574-5p
let-7c-5p	miR-153-5p	miR-3689b-3p	miR-609
let-7d-5p	miR-188-3p	miR-3689c	miR-6513-3p
let-7e-5p	miR-19a-3p	miR-3975	miR-6776-5p
let-7f-5p	miR-19b-1-5p	miR-4429	miR-6779-5p
let-7g-5p	miR-19b-3p	miR-4458	miR-6780a-5p
let-7i-5p	miR-202-3p	miR-4520-5p	miR-6785-5p
miR-103a-1-5p	miR-222-3p	miR-4538	miR-6799-5p
miR-12124	miR-223-5p	miR-454-3p	miR-6872-5p
miR-122-5p	miR-297	miR-4659b-3p	miR-6873-3p
miR-124-3p	miR-301a-3p	miR-4728-5p	miR-6883-5p
miR-1273h-5p	miR-301b-3p	miR-4738-3p	miR-7106-5p
miR-1288-5p	miR-30b-3p	miR-490-3p	miR-7161-5p
miR-130a-3p	miR-3127-5p	miR-5011-3p	miR-766-3p
miR-130b-3p	miR-3129-5p	miR-501-5p	miR-8087
miR-132-3p	miR-3161	miR-508-5p	miR-9-5p
miR-143-5p	miR-320a-3p	miR-5100	miR-9718
miR-148a-3p	miR-320b	miR-513a-5p	miR-98-5p
miR-148b-3p	miR-320c	miR-542-5p	

Table S7. The 131 upregulated circRNAs and name conversion results from the GSE101850 dataset.

GPL19978_ID	circBase_ID	GPL19978_ID	circBase_ID	GPL19978_ID	circBase_ID
circRNA_103420	circ_0066631	circRNA_102540	circ_0006670	circRNA_104375	circ_0080212
circRNA_103433	circ_0005332	circRNA_104633	circ_0084606	circRNA_102368	circ_0047663
circRNA_103433	circ_0005332	circRNA_104633	circ_0084606	circRNA_101372	circ_0006278
circRNA_103421	circ_0004968	circRNA_103798	circ_0005260	circRNA_100346	circ_0004717
circRNA_101002	circ_0025201	circRNA_103350	circ_0065220	circRNA_100570	circ_0001963
circRNA_102092	circ_0044177	circRNA_101356	circ_0004846	circRNA_102515	circ_0008033
circRNA_101003	circ_0025202	circRNA_104789	circ_0087232	circRNA_102769	circ_0008440
circRNA_104268	circ_0078738	circRNA_104614	circ_0005847	circRNA_104039	circ_0075402
circRNA_001038	circ_0000453	circRNA_400097	circ_0092319	circRNA_104304	circ_0079363
circRNA_100679	circ_0005620	circRNA_102471	circ_0000907	circRNA_100184	circ_0000061
circRNA_104373	circ_0080209	circRNA_103066	circ_0006332	circRNA_100157	circ_0011462
circRNA_104797	circ_0087293	circRNA_104337	circ_0079672	circRNA_104795	circ_0087283
circRNA_100799	circ_0006988	circRNA_103291	circ_0006673	circRNA_100487	circ_0005223
circRNA_104372	circ_0008334	circRNA_102450	circ_0006877	circRNA_103439	circ_0001330
circRNA_102595	circ_0052095	circRNA_103004	circ_0006612	circRNA_105007	circ_0091024
circRNA_104791	circ_0087234	circRNA_101414	circ_0032746	circRNA_104301	circ_0006093
circRNA_104798	circ_0087300	circRNA_101159	circ_0002857	circRNA_102810	circ_0056285
circRNA_103087	circ_0060904	circRNA_102646	circ_0008257	circRNA_102512	circ_0050463
circRNA_104038	circ_0075393	circRNA_102837	circ_0008010	circRNA_100150	circ_0011424
circRNA_104970	circ_0002172	circRNA_103123	circ_0002360	circRNA_102897	circ_0003747
circRNA_101316	circ_0000523	circRNA_104338	circ_0079673	circRNA_102001	circ_0005415
circRNA_100665	circ_0019589	circRNA_101846	circ_0003220	circRNA_101081	circ_0000408
circRNA_100664	circ_0005741	circRNA_102230	circ_0046215	circRNA_102481	circ_0003253
circRNA_101592	circ_0036287	circRNA_100486	circ_0001958	circRNA_104094	circ_0076054
circRNA_101666	circ_0037130	circRNA_103233	circ_0008360	circRNA_100648	circ_0005898

circRNA_102480	circ_0049965	circRNA_101816	circ_0005941	circRNA_102964	circ_0005059
circRNA_100195	circ_0012144	circRNA_102914	circ_0058189	circRNA_101462	circ_0034168
circRNA_100666	circ_0019591	circRNA_101082	circ_0002588	circRNA_100100	circ_0010931
circRNA_104339	circ_0079675	circRNA_100491	circ_0003314	circRNA_102468	circ_0002259
circRNA_101174	circ_0028864	circRNA_100099	circ_0003757	circRNA_104469	circ_0082140
circRNA_002149	circ_0001627	circRNA_103706	circ_0002782	circRNA_101781	circ_0006012
circRNA_100354	circ_0014613	circRNA_101083	circ_0027033	circRNA_102804	circ_0056139
circRNA_104781	circ_0087063	circRNA_000212	circ_0000705	circRNA_103297	circ_0064416
circRNA_103020	circ_0001137	circRNA_102561	circ_0051246	circRNA_104935	circ_0003362
circRNA_103124	circ_0001187	circRNA_103187	circ_0005175	circRNA_102954	circ_0003923
circRNA_103086	circ_0060903	circRNA_400085	circ_0092334	circRNA_104241	circ_0004019
circRNA_102469	circ_0008432	circRNA_101720	circ_0002078	circRNA_104115	circ_0005451
circRNA_100667	circ_0000255	circRNA_103351	circ_0065223	circRNA_101821	circ_0007079
circRNA_104156	circ_0001626	circRNA_100981	circ_0024737	circRNA_104137	circ_0076995
circRNA_104470	circ_0082141	circRNA_101084	circ_0027035	circRNA_101327	circ_0031361
circRNA_104913	circ_0002544	circRNA_102555	circ_0002882	circRNA_103210	circ_0004470
circRNA_104468	circ_0082139	circRNA_103208	circ_0063019	circRNA_102488	circ_0005568
circRNA_102924	circ_0008365	circRNA_104519	circ_0004351	circRNA_102819	circ_0056433
circRNA_100245	circ_0005354	circRNA_102071	circ_0003530		
